# Early detection of the major male cancer types in blood-based liquid biopsies using a DNA methylation panel

**DOI:** 10.1186/s13148-019-0779-x

**Published:** 2019-12-02

**Authors:** Vera Constâncio, Sandra P. Nunes, Catarina Moreira-Barbosa, Rui Freitas, Jorge Oliveira, Inês Pousa, Júlio Oliveira, Marta Soares, Carlos Gonçalves Dias, Teresa Dias, Luís Antunes, Rui Henrique, Carmen Jerónimo

**Affiliations:** 1 Cancer Biology & Epigenetics Group—Research Center, Portuguese Oncology Institute of Porto (CI-IPOP), LAB 3, F Bdg, 1st floor Rua Dr. António Bernardino de Almeida, 4200-072 Porto, Portugal; 2 Master in Oncology, Institute of Biomedical Sciences Abel Salazar—University of Porto (ICBAS-UP), Rua de Jorge Viterbo Ferreira no. 228, 4050-313 Porto, Portugal; 3 Urology Clinic, Portuguese Oncology Institute of Porto, Rua Dr. António Bernardino de Almeida, 4200-072 Porto, Portugal; 4 Lung Cancer Clinic and Department of Medical Oncology, Portuguese Oncology Institute of Porto, Rua Dr. António Bernardino de Almeida, 4200-072 Porto, Portugal; 5 Digestive Tract Pathology Clinic and Surgical Oncology, Portuguese Oncology Institute of Porto, Rua Dr. António Bernardino de Almeida, 4200-072 Porto, Portugal; 6 Department of Epidemiology, Portuguese Oncology Institute of Porto, Rua Dr. António Bernardino de Almeida, 4200-072 Porto, Portugal; 7 Department of Pathology, Portuguese Oncology Institute of Porto, Rua Dr. António Bernardino de Almeida, 4200-072 Porto, Portugal; 8 Department of Pathology and Molecular Immunology, Institute of Biomedical Sciences Abel Salazar—University of Porto (ICBAS-UP), Rua de Jorge Viterbo Ferreira no. 228, 4050-313 Porto, Portugal

**Keywords:** Lung cancer, Prostate cancer, Colorectal cancer, Epigenetic biomarkers, DNA methylation, Circulating cell free DNA, Liquid biopsy

## Abstract

**Background:**

Lung (LC), prostate (PCa) and colorectal (CRC) cancers are the most incident in males worldwide. Despite recent advances, optimal population-based cancer screening methods remain an unmet need. Due to its early onset, cancer specificity and accessibility in body fluids, aberrant DNA promoter methylation might be a valuable minimally invasive tool for early cancer detection. Herein, we aimed to develop a minimally invasive methylation-based test for simultaneous early detection of LC, PCa and CRC in males, using liquid biopsies.

**Results:**

Circulating cell-free DNA was extracted from 102 LC, 121 PCa and 100 CRC patients and 136 asymptomatic donors’ plasma samples. Sodium-bisulfite modification and whole-genome amplification was performed. Promoter methylation levels of *APC*_*me*_*, FOXA1*_*me*_*, GSTP1*_*me*_*, HOXD3*_*me*_, *RARβ2*_*me*_*, RASSF1A*_*me*_*, SEPT9*_*me*_ and *SOX17*_*me*_ were assessed by multiplex quantitative methylation-specific PCR.

*SEPT9*_*me*_ and *SOX17*_*me*_ were the only biomarkers shared by all three cancer types, although they detected CRC with limited sensitivity. A “PanCancer” panel (*FOXA1*_*me*_*, RARβ2*_*me*_ and *RASSF1A*_*me*_) detected LC and PCa with 64% sensitivity and 70% specificity, complemented with “CancerType” panel (*GSTP1*_*me*_ and *SOX17*_*me*_) which discriminated between LC and PCa with 93% specificity, but with modest sensitivity. Moreover, a *HOXD3*_*me*_ and *RASSF1A*_*me*_ panel discriminated small cell lung carcinoma from non-small cell lung carcinoma with 75% sensitivity, 88% specificity, 6.5 LR+ and 0.28 LR–. An *APC*_*me*_ and *RASSF1A*_*me*_ panel independently predicted disease-specific mortality in LC patients.

**Conclusions:**

We concluded that a DNA methylation-based test in liquid biopsies might enable minimally invasive screening of LC and PCa, improving patient compliance and reducing healthcare costs. Moreover, it might assist in LC subtyping and prognostication.

## Background

According to GLOBOCAN, cancers of the lung (LC), prostate (PCa), and colorectal (CRC) were estimated to account, globally, for 39% of all cancers diagnosed in males worldwide in 2018, or 46% if only Europe was considered [1], representing major public health issues. Despite advances in therapeutic strategies over the years, mortality rates remain high, mainly due to late diagnosis. Hence, the development and implementation of effective screening methods that might allow for easy detection of these cancers at early stages, when treatment is most likely to be curative, is crucial. Although no LC screening method is currently implemented, low-dose computed tomography (LD-CT) has been suggested owing to a reduction in LC-related mortality in screening trials targeting high-risk smokers [2–4], although at the expense of a high rate of false-positive results [2, 5]. The widespread adoption of serum prostate-specific antigen (PSA) and digital rectal examination (DRE) based PCa screening has increased the detection of early stage disease [6]. However, serum PSA-based screening is associated with significant overdiagnosis and overtreatment of non-life threatening PCa [7], whereas DRE results are dependent on clinicians’ expertise and mostly detects advanced-stage disease [6]. For CRC screening, both faecal occult blood test (FOBT) and coloscopy-based screening are available options [8]. Nonetheless, the former has limited sensitivity to detect precancerous lesions, and although colonoscopy is very precise and enables resection of precancerous polyps during examination, it is an invasive and expensive procedure with low patient compliance [9]. Although the aforementioned strategies might help decrease cancer-related mortality among those three cancer types, considering the limitations, potential harms for men’s quality of life and the negative economic impact in healthcare systems, more effective and minimally invasive methods are required.

Aberrant DNA promoter methylation, which is closely associated with inappropriate gene transcription and is thought to precede the emergence of the malignant phenotype, can be easily detected with minimal-invasiveness in circulating cell-free DNA (ccfDNA) from body fluids, such as serum/plasma [10–13], representing a valuable tool for early cancer detection. Several studies have assessed the feasibility of ccfDNA methylation for specific cancer type detection [13, 14], including “Epi proColon” (*SEPT9*_*me*_) and “Epi proLung” (*PTGER4*_*me*_ and *SHOX2*_*me*_), two commercially available tests for CRC and LC detection, respectively [15, 16]. Nonetheless, to the best of our knowledge, only few research teams have investigated the feasibility of this approach for simultaneous detection of several types of cancer [17–20]. Herein, we sought to determine the feasibility of using a minimally invasive methylation-based test (comprising eight candidate genes selected based on a literature review) in liquid biopsies for simultaneous detection of LC, PCa and CRC in males. Additionally, the prognostic value of the selected genes was also tested.

## Results

### Clinical and pathological data

Plasma samples were obtained from 323 male patients diagnosed with LC (*n* = 102), PCa (*n* = 121) and CRC (*n* = 100), and 136 male AC (Table 1). Overall, cancer patients’ median age was significantly higher than that of controls (*p* < 0.0001). Nonetheless, except for *SOX17*_*me*_ levels that correlated with controls’ age (*R* = 0.179; *p* = 0.037), and *FOXA1*_*me*_ levels which correlated with cancer patients’ age (*R* = 0.144; *p* = 0.010), no other correlations were disclosed.

**Table 1:** Clinical and pathological features of LC, PCa and CRC patient and ACs included in this study

Clinicopathological features	AC	Cancer patients
Number	136	323
Age median (range)	57 (48–66)	68 (27–93)
Lung cancer		
Number		102
Age median (range)		66 (45–89)
Histological type	n.a.	
Non-small cell lung carcinoma (NSCLC)		
Adenocarcinoma		42
Squamous cell carcinoma		43
Large cell carcinoma		1
Small cell lung carcinoma (SCLC)		16
Primary tumour (T)^a^	n.a.	
T1		12
T2/T3/T4		82
Regional lymph node (N)^b^	n.a.	
N0		25
N+		72
Distant metastasis (M)	n.a.	
M0		47
M+		55
Clinical stage	n.a.	
I/II		17
III/IV		85
Prostate cancer		
Number		121
Age median (range)		71 (52–88)
Histological type	n.a.	
Adenocarcinoma		121
Primary tumour (T)^c^	n.a.	
T1/T2		104
T3		16
Regional lymph node (N)	n.a.	
N0		119
N+		2
Distant metastasis (M)	n.a.	
M0		116
M+		5
Grade group	n.a.	
1		59
2		38
3/4/5		24
Serum PSA levels (ng/mL)	n.a.	
< 10		71
10–20		27
> 20		23
Clinical stage	n.a.	
I		31
II		55
III/IV		35
Colorectal cancer		
Number		100
Age median (range)		66 (27–93)
Histological Type	n.a.	
Adenocarcinoma (all subtypes)		99
Squamous cell carcinoma		1
Tumour location	n.a.	
Proximal colon		23
Distal colon		36
Rectum		41
Primary tumour (T)^d^	n.a.	
T1/T2		26
T3/T4		72
Regional lymph node (N)^e^	n.a.	
N0		40
N+		57
Distant metastasis (M)	n.a.	
M0		82
M+		18
Clinical stage	n.a.	
I/II		39
III/IV		61

^a^No information available in 8 cases

^b^No information available in 5 cases

^c^No information available in 1 case

^d^No information available in 2 cases

^e^No information available in 3 cases

*n.a*. not applicable

### Gene promoter methylation levels in ccfDNA

**Fig. 1:**
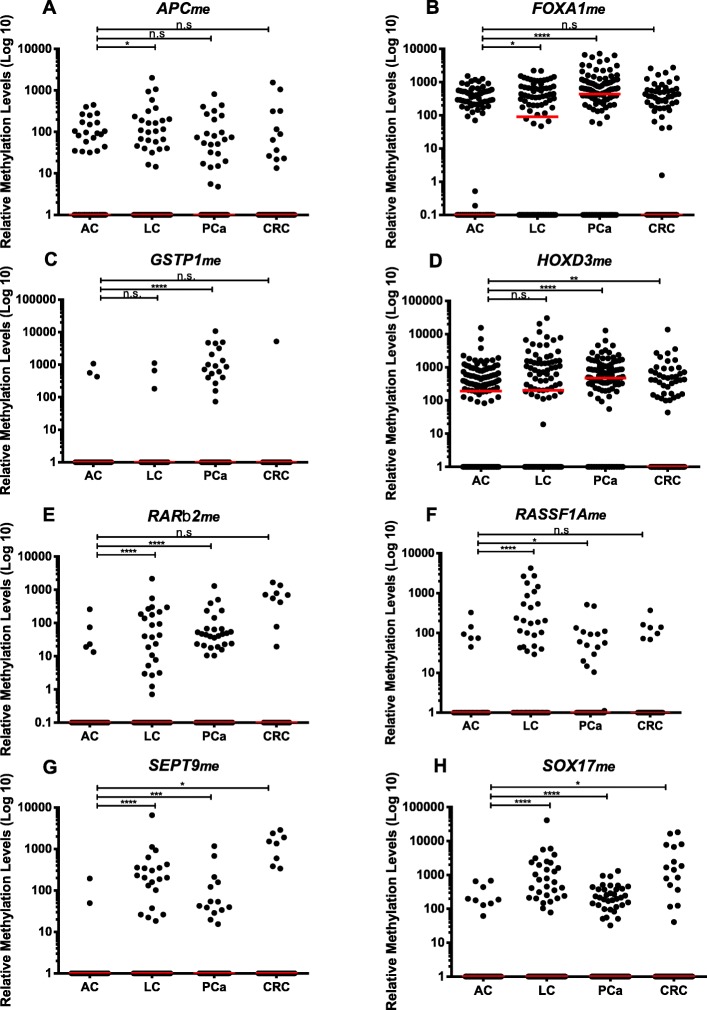
Distribution of **a** *APC*, **b** *FOXA1*, **c** *GSTP1*, **d** *HOXD3*, **e** *RARβ2*, **f** *RASSF1A*, **g** *SEPT9* and **h** *SOX17* relative methylation levels of asymptomatic controls (AC) (n = 136), lung cancer (LC) (n = 102), prostate cancer (PCa) (n = 121) and colorectal cancer (CRC) (n = 100) samples. Mann-Whitney U Test between AC and each cancer type, n.s. *p* > 0.05, *p < 0.05, **p < 0.01, ***p < 0.001, ****p < 0.0001. Red horizontal lines represent median methylation levels

CcfDNA’s *APC*_*me*_*, FOXA1*_*me*_*, GSTP1*_*me*_*, HOXD3*_*me*_*, RARβ2*_*me*_*, RASSF1A*_*me*_*, SEPT9*_*me*_ and *SOX17*_*me*_ levels were compared between each cancer type and controls. *APC*_*me*_ (*p* = 0.033), *FOXA1*_*me*_ (*p* = 0.024), *RARβ2*_*me*_, *RASSF1A*_*me*_, *SEPT9*_*me*_ and *SOX17*_*me*_ (*p* < 0.0001) levels were significantly higher in LC patients than in AC, whereas no differences were disclosed for *GSTP1*_*me*_ and *HOXD3*_*me*_ (*p* = 0.718 and *p* = 0.174, respectively) (Fig. 1).

In PCa patients, significantly higher levels were observed for *FOXA1*_*me*_*, GSTP1*_*me*_*, HOXD3*_*me*_*, RARβ2*_*me*_*, SOX17*_*me*_ (*p* < 0.0001), *RASSF1A*_*me*_ and *SEPT9*_*me*_ (*p* = 0.014 and *p* = 0.0001, respectively), although no differences were apparent for *APC*_*me*_ (*p* = 0.443) (Fig. 1).

Concerning CRC patients, only *SEPT9*_*me*_ and *SOX17*_*me*_ (*p* = 0.012 and *p* = 0.014, respectively) displayed significantly higher levels in patients comparing with controls. Moreover, *HOXD3*_*me*_ levels (*p* = 0.009) were significantly lower in CRC patients than in controls (Fig. 1).

### Biomarker performance of ccfDNA

Since our main goal was to test biomarker performance for LC, PCa and CRC detection of a methylation-based panel in ccfDNA, genes displaying significantly higher methylation levels in cancer patients compared to controls were selected for further analysis.

*SEPT9*_*me*_ and *SOX17*_*me*_ were the only two biomarkers shared by all three cancer types, displaying specificity between 93 and 100% (Table 2). Nonetheless, these two biomarkers detected CRC with limited sensitivity (8 and 11%, respectively) (Table 2), thus, no further analyses were performed for this tumour.

**Table 2:** Biomarker performance of each gene promoter methylation for LC, PCa and CRC detection in ccfDNA

Genes	AUC (95 % CI)	*p*-value	Cut-off value	Sensitivity % (95% CI)	Specificity % (95% CI)	Accuracy % (95% CI)	Positive likelihood ratio (LR+) (95% CI)	Negative likelihood ratio (LR-) (95% CI)
	Lung Cancer							
*APC*_*me*_	0.556 (0.482-0.631)	0.136	7.2629	26 (18-36)	85 (77-90)	60 (53-66)	1.7 (1.0-2.9)	0.87 (0.76-1.00)
*FOXA1*_*me*_	0.579 (0.505-0.653)	0.037	301.1682	38 (29-48)	77 (69-84)	61 (54-67)	1.7 (1.1-2.5)	0.80 (0.67-0.96)
*RARβ2*_*me*_	0.599 (0.525-0.674)	0.009	0.3548	24 (16-33)	96 (92-99)	65 (59-71)	6.4 (2.5-16.2)	0.79 (0.71-0.89)
*RASSF1A*_*me*_	0.602 (0.528-0.677)	0.007	14.5588	25 (17-34)	96 (91-98)	65 (59-71)	5.6 (2.4-13.0)	0.79 (0.70-0.89)
*SEPT9*_*me*_	0.596 (0.522-0.671)	0.011	9.1907	21 (13-30)	99 (95-100)	65 (59-71)	14.0 (3.4-58.4)	0.81 (0.73-0.89)
*SOX17*_*me*_	0.619 (0.546-0.693)	0.002	70.0725	29 (21-39)	94 (89-97)	66 (60-72)	5.0 (2.4-10.4)	0.75 (0.66-0.86)
LC panel	-	-	-	66 (56-75)	70 (61-77)	68 (62-74)	2.2 (1.6-2.9)	0.49 (0.37-0.66)
	Prostate Cancer							
*FOXA1*_*me*_	0.719 (0.656-0.782)	<0.001	295.8133	61 (52-70)	77 (69-84)	70 (64-75)	2.7 (1.9-3.8)	0.50 (0.40-0.64)
*GSTP1*_*me*_	0.564 (0.493-0.634)	0.078	36.7086	15 (9-22)	98 (94-100)	59 (52-65)	6.7 (2.0-22.3)	0.87 (0.80-0.94)
*HOXD3*_*me*_	0.650 (0.583-0.717)	<0.001	320.9365	80 (72-87)	43 (34-51)	60 (54-66)	1.4 (1.2-1.7)	0.47 (0.31-0.70)
*RARβ2*_*me*_	0.594 (0.524-0.664)	0.010	5.2251	22 (15-31)	96 (92-99)	61 (55-67)	6.1 (2.4-15.3)	0.81 (0.73-0.89)
*RASSF1A*_*me*_	0.543 (0.472-0.614)	0.232	0.5627	13 (8-21)	96 (91-98)	57 (51-64)	3.0 (1.2-7.4)	0.91 (0.84-0.98)
*SEPT9*_*me*_	0.550 (0.479-0.621)	0.164	7.7096	12 (6-19)	99 (95-100)	58 (51-64)	7.9 (1.8-33.9)	0.90 (0.84-0.96)
*SOX17*_*me*_	0.611 (0.542- 0.681)	0.002	16.153	29 (21-38)	93 (88-97)	63 (57-69)	4.4 (2.2-8.7)	0.76 (0.67-0.86)
PCa panel	-	-	-	72 (63-80)	72 (64-79)	72 (66-77)	2.6 (1.9-3.5)	0.39 (0.29-0.53)
	Colorectal cancer							
*SEPT9*_*me*_	0.533 (0.458 - 0.608)	0.383	265.2606	8 (4-15)	100 (97-100)	61 (54-67)	-	0.92 (0.87-0.97)
*SOX17*_*me*_	0.550 (0.475 - 0.625)	0.189	732.3866	11 (6-19)	100 (97-100)	62 (56-68)	-	0.89 (0.83-0.95)
CRC panel	-	-	---	12 (6-20)	100 (97-100)	63 (56-69)	-	0.88 (0.82-0.95)

LC panel—*FOXA1*_*me*_*, RARβ2*_*me*_*, RASSF1A*_*me*_ and *SOX17*_*me*_; PCa panel—*FOXA1*_*me*_*, RARβ2*_*me*_*, RASSF1A*_*me*_ and *GSTP1*_*me*_; CRC panel—*SEPT9*_*me*_ and *SOX17*_*me*_

*RARβ2*_*me*_*, RASSF1A*_*me*_*, SEPT9*_*me*_ *and SOX17*_*me*_ were able to detect both LC and PCa with over 93% specificity (Table 2). Conversely, *FOXA1*_*me*_ disclosed the highest sensitivity for detecting LC and the second highest for detecting PCa (38% and 61%, respectively), with 77% specificity for both. *SOX17*_*me*_ detected both LC and PCa, individually, with 29% sensitivity, and *RARβ2*_*me*_ identified both cancers with 22–24% sensitivity (Table 2).

Gene panels were further constructed to increase detection sensitivity. Hence, the best LC panel (*FOXA1*_*me*_*, RARβ2*_*me*_*, RASSF1A*_*me*_ and *SOX17*_*me*_) achieved 66% sensitivity and 70% specificity, whereas for PCa, the panel (*FOXA1*_*me*_*, RARβ2*_*me*_*, RASSF1A*_*me*_ and *GSTP1*_*me*_) depicted 72% sensitivity and specificity (Table 2).

**Fig. 2:**
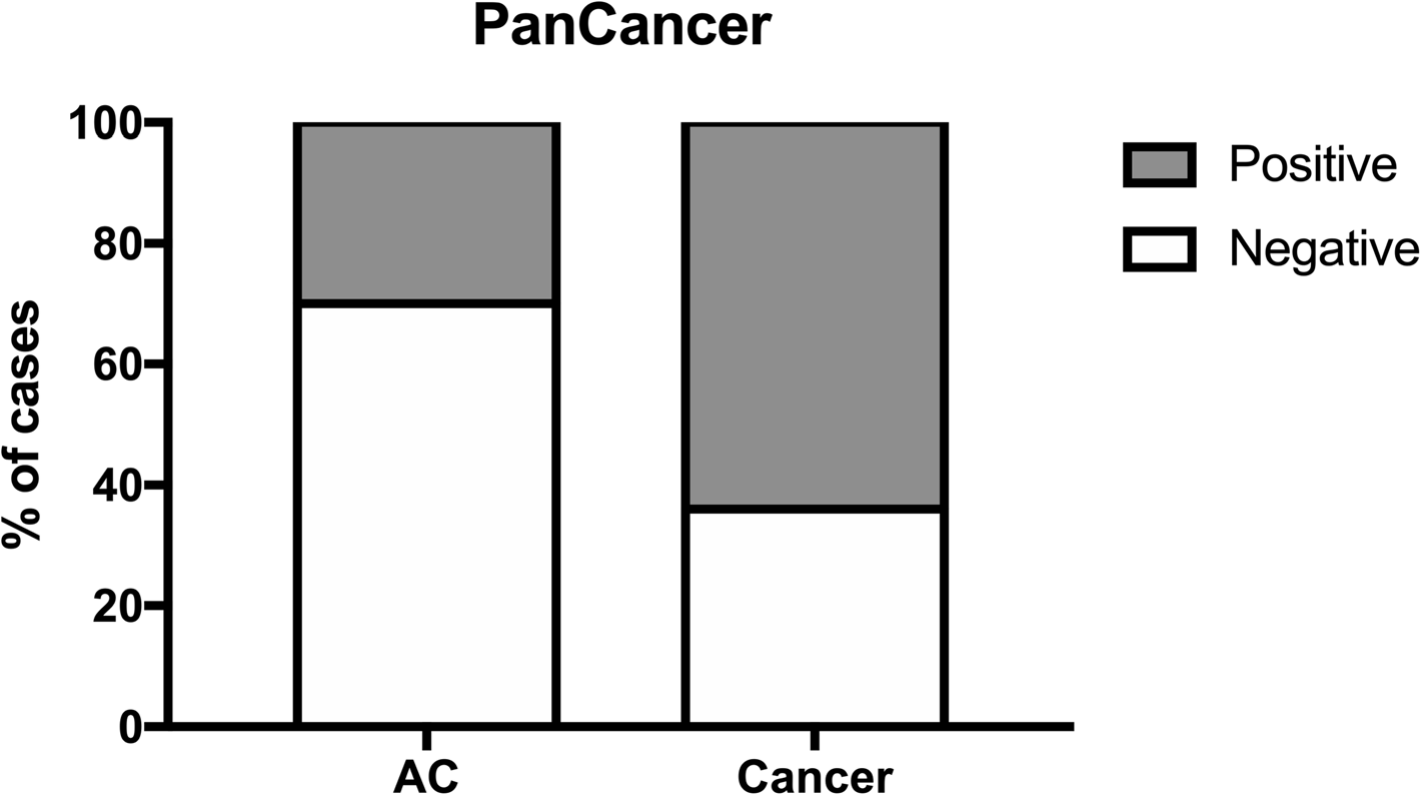
Percentage of cases identified by “PanCancer” panel in cancer samples (64% positive, 36% negative) and in asymptomatic controls (ACs) (30% positive, 70% negative)

Aiming to obtain a gene panel for simultaneous LC and PCa detection (designated as “PanCancer” panel), all genes (*FOXA1*_*me*_, *RARβ2*_*me*_, *RASSF1A*_*me*_ and *SEPT9*_*me*_) that were shared [except for *SOX17*_*me*_ that displayed a relatively different cut-off between these two cancer types (70.0725 for LC and 16.153 for PCa)) (Table 2), were further tested as panel. Additionally, *APC*_*me*_, *GSTP1*_*me*_, *HOXD3*_*me*_ and *SOX17*_*me*_ were tested as a suitable panel for tumour’s primary location discrimination (“CancerType” panel). Remarkably, the “PanCancer” panel (*FOXA1*_*me*_, *RARβ2*_*me*_ and *RASSF1A*_*me*_) identified LC and PCa with 64% sensitivity, 70% specificity, 66% accuracy, positive likelihood ratio (LR+) of 2.3 and a negative likelihood ratio (LR–) of 0.53 (Table 3 and Fig. 2).

**Table 3:** Biomarker performance detection of “PanCancer” panel *(FOXA1*_*me*_*, RARβ2*_*me*_ and *RASSF1A*_*me*_) in ccfDNA

	PanCancer
Sensitivity %	64.3
Specificity %	69.8
Accuracy %	66.4
Positive likelihood ratio (LR+)	2.3
Negative likelihood ratio (LR-)	0.52

As early diagnosis is imperative and specifically for PCa it is also relevant to detect clinically significant disease, we further tested this panel for those purposes. “PanCancer” panel detected LC stages I and II with 95% (95% CI 90–98%) specificity, however with modest 35% (95% CI 14–62%) sensitivity, displaying 88% (95% CI 81–93%) accuracy, LR+ of 7.1 (95% CI 2.6–19.6) and LR– of 0.68 (95% CI 0.48–0.97). Importantly, this panel was able to discriminate intermediate and high-risk PCa patients (stage II and III-IV) from controls and low-risk (stage I) PCa patients with 71% (95% CI 61–80%) sensitivity, 65% (95% CI 57–72%) specificity, 67% (61–73%) accuracy, LR+ of 2.0 (1.6–2.6) and LR– of 0.45 (0.32–0.63).

Among the four genes tested for “CancerType” panel, *SOX17*_*me*_ and *GSTP1*_*me*_ demonstrated the best performance for discriminating LC from PCa. Indeed, *SOX17*_*me*_ discriminated LC from PCa with 93% specificity, whereas *GSTP1*_*me*_ detected PCa with 97% specificity, although with limited sensitivity (Table 4).

**Table 4:** Biomarker performance of “CancerType” for discrimination among LC and PCa

Gene	Sensitivity %	Specificity %	Accuracy %	Positive likelihood ratio (LR+)	Negative likelihood ratio (LR–)
Lung cancer					
*SOX17*_*me*_	15.2	93.4	57.7	3.0	0.91
*GSTP1*_*me*_	---	---	---	---	---
Prostate cancer					
*SOX17*_*me*_	---	---	---	---	---
*GSTP1*_*me*_	13.6	97.0	51.8	3.6	0.89

### Association between promoters’ methylation levels and clinicopathological features

**Fig. 3:**
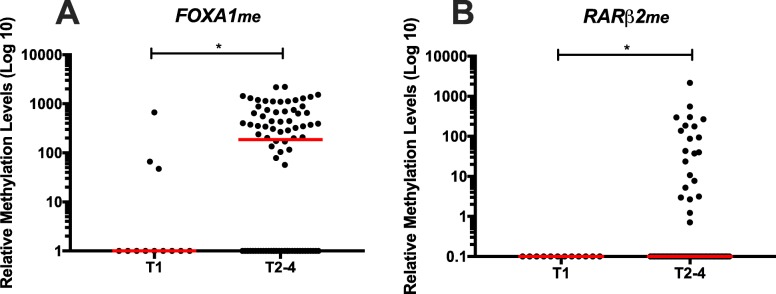
**a** *FOXA1* and **b** *RARβ2* promoter’s methylation levels according with T stage (T1 (n = 12) and T2-4 (n = 82)) in LC patients. Mann-Whitney U Test, *p < 0.05. Red horizontal lines represent median methylation levels

**Fig. 4:**
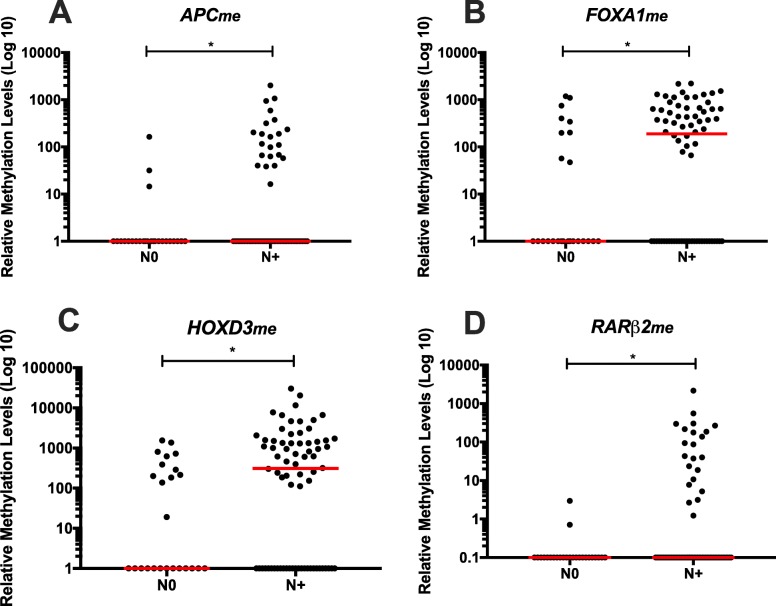
**a** *APC*, **b** *FOXA1*, **c** *HOXD3* and **d** *RARβ2* promoter’s methylation levels according with node status, node-negative (N0) (n = 25) and node-positive (N+) (n = 72) in LC patients. Mann-Whitney U Test, *p < 0.05. Red horizontal lines represent median methylation levels

Concerning associations between promoters’ methylation levels and clinicopathological features (Additional file 1: Tables S1–S3), in LC patients, higher circulating *FOXA1*_*me*_ and *RARβ2*_*me*_ levels associated with advanced primary tumour stage (T) (*p* = 0.014 and *p* = 0.044, respectively) (Fig. 3), whereas higher levels of those two genes and *APC*_*me*_ and *HOXD3*_*me*_ were also depicted in LC patients with regional lymph node involvement (*p* = 0.021, *p* = 0.015, *p* = 0.044 and *p* = 0.022, respectively) (Fig. 4).

**Fig. 5:**
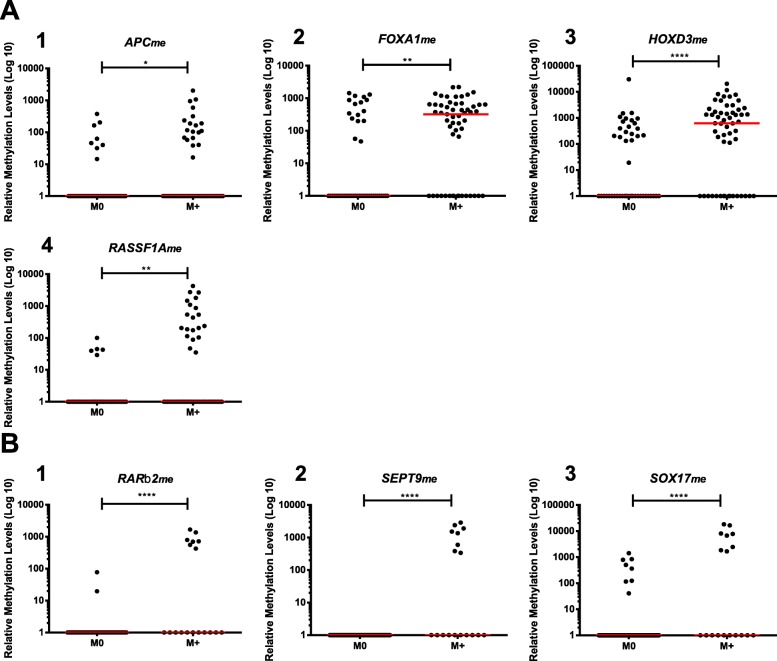
Distribution of methylation levels in LC **a** and in CRC patients according with metastatic dissemination **b**. **a** (1) *APC*, (2) *FOXA1*, (3) *HOXD3* and (4) *RASSF1A* promoter’s methylation levels in non-metastatic (M0) (n = 47) and metastatic (M+) (n = 55) LC patients. **b** (1) *RARβ2*, (2) *SEPT9* and (3) *SOX17* promoter’s methylation levels in non-metastatic (M0) (n = 82) and metastatic (M+) (n = 18) CRC patients. Mann-Whitney U Test, *p < 0.05, **p < 0.01, ****p < 0.0001. Red horizontal lines represent median methylation levels

Furthermore, circulating *APC*_*me*_*, FOXA1*_*me*_*, HOXD3*_*me*_ and *RASSF1A*_*me*_ levels were significantly higher in LC patients with distant metastatic disease (*p* = 0.028, *p* = 0.001, *p* < 0.0001 and *p* = 0.001, respectively) (Fig. 5a), whereas *RARβ2*_*me*_, *SEPT9*_*me*_ and *SOX17*_*me*_ levels were higher in CRC metastatic patients (*p* < 0.0001, in all comparisons) (Fig. 5b). Similarly, significantly higher *APC*_*me*_*, GSTP1*_*me*_*, HOXD3*_*me*_, *RARβ2*_*me*_, *RASSF1A*_*me*_ and *SEPT9*_*me*_ levels were observed in the five PCa patients with metastatic disease at diagnosis (*p* < 0.0001, for all comparisons, except for *HOXD3*_*me*_, *p* = 0.003) (Additional file 2: Figure S1).

**Fig. 6:**
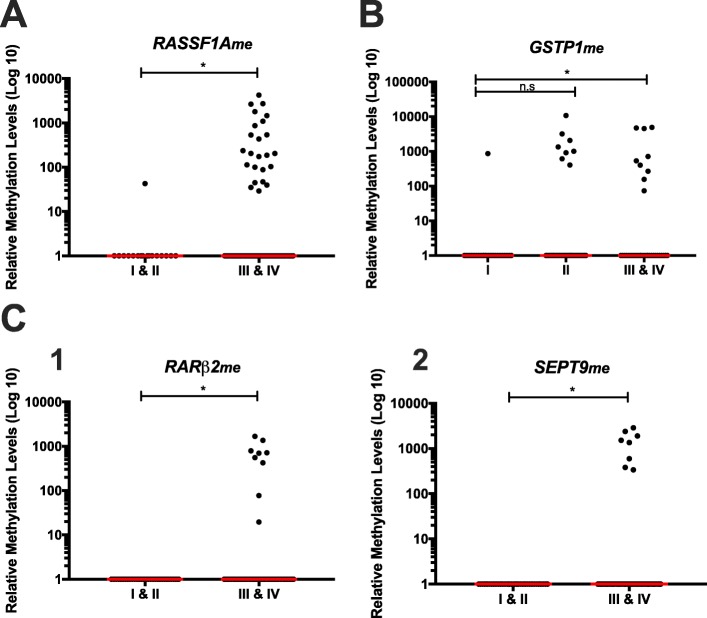
Scatter plot of **a** *RASSF1A* promoter methylation levels between clinical stage I & II (n = 17) and III & IV (n = 85) LC patients. **b** *GSTP1* promoter methylation levels between clinical stage I (n = 31), II (n = 55) and III & IV (n = 35) PCa patients. **c** (1) *RARβ2* and (2) *SEPT9* promoters’ methylation levels between clinical stage I & II (n = 39) and III & IV (n = 61) CRC patients. Mann-Whitney U Test for **a** and **c** and Mann-Whitney U test with Bonferroni’s correction for **b**, n.s. *p* < 0.05, *p < 0.05. Red horizontal lines represent median methylation

Significantly higher circulating *RASSF1A*_*me*_ levels were also apparent in LC patients with advanced clinical stage (III and IV) (*p* = 0.043) (Fig. 6a), whereas higher *GSTP1*_*me*_ levels were observed in PCa patients with advanced clinical stage (III and IV) (*p* = 0.039) comparing to patients with clinical stage I (Fig. 6b). Similar results were found for *RARβ2*_*me*_ and *SEPT9*_*me*_ in CRC patients (*p* = 0.012 and *p* = 0.019, respectively) (Fig. 6c).

**Fig. 7:**
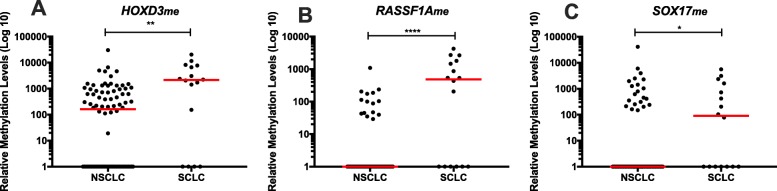
Scatter plot of **a** *HOXD3*, **b** *RASSF1A*, and **c** *SOX17* promoter’s methylation levels according with histological subtype (Non-small cell lung carcinoma (NSCLC) (n = 86) and small cell lung carcinoma (SCLC) (n = 16)). Mann-Whitney U Test, *p < 0.05, **p < 0.01, ****p < 0.0001. Red horizontal lines represent median methylation levels

Concerning LC patients, significantly higher circulating *HOXD3*_*me*_*, RASSF1A*_*me*_ and *SOX17*_*me*_ levels were observed in small cell lung cancer (SCLC) vs non-small cell lung cancer (NSCLC) patients (*p* = 0.001, *p* < 0.001 and *p* = 0.013, respectively) (Fig. 7). Therefore, we further assessed biomarker’s performance of these three genes to discriminate between these two major histological subtypes (Table 5). Individually, all three genes identified SCLC with specificities above 75%. *RASSF1A*_*me*_ disclosed the highest specificity (98% (95% CI 92–100%)), correctly identifying 10 out of the 16 SCLC patients, misclassifying only 2 out of 86 NSCLC patients. Importantly, the panel including the two genes with the highest specificity (*HOXD3*_*me*_ and *RASSF1A*_*me*_) discriminated between LC subtypes with 75% (95% CI 48–93%) sensitivity, 88% (95% CI 80–94%) specificity, 86% (95% CI 78–92%) accuracy, LR+ of 6.5 (95% CI 3.4–12.3) and LR– of 0.28 (95% CI of 0.12–0.66) (Table 5).

**Table 5:** Biomarker performance of *HOXD3*, *RASSF1A* and *SOX17* promoters’ methylation levels for discrimination among small cell lung carcinoma (SCLC) and non-small cell Lung carcinoma (NSCLC)

Genes	AUC (95 % CI)	*p*-value	Cut-off value	Sensitivity % (95% CI)	Specificity % (95% CI)	Accuracy % (95% CI)	Positive likelihood ratio (LR+) (95% CI)	Negative likelihood ratio (LR–) (95% CI)
SCLC vs NSCLC								
*HOXD3*_*me*_	0.742 (0.577–0.907)	*0.002*	1401.1301	69 (41–89)	88 (80–94)	85 (77–92)	5.9 (3.0–11.6)	0.35 (0.17–0.73)
*RASSF1A*_*me*_	0.775 (0.617–0.934)	*< 0.001*	204.8474	63 (35–85)	98 (92–100)	92 (85–97)	26.9 (6.5–113.3)	0.38 (0.20–0.72)
*SOX17*_*me*_	0.657 (0.504–0.811)	*0.046*	30.1876	56 (30–80)	76 (65–84)	73 (63–81)	2.3 (1.3–4.1)	0.58 (0.33–1.02)
*HOXD3*_*me*_*/RASSF1A*_*me*_	-	-	-	75 (48–93)	88 (80–94)	86 (78–92)	6.5 (3.4–12.3)	0.28 (0.12–0.66)

### Prognostic biomarker performance of ccfDNA

The median patient follow-up time for LC patients’ cohort was 9 months (range from 0 to 40 months), during which 62 patients have deceased due to cancer. Since only 15 out of 102 LC patients were submitted to surgery, disease-free survival (DFS) was not assessed. Moreover, no significant associations were disclosed between circulating methylation levels of the selected genes and progression-free survival (PFS) (data not shown).

**Fig. 8:**
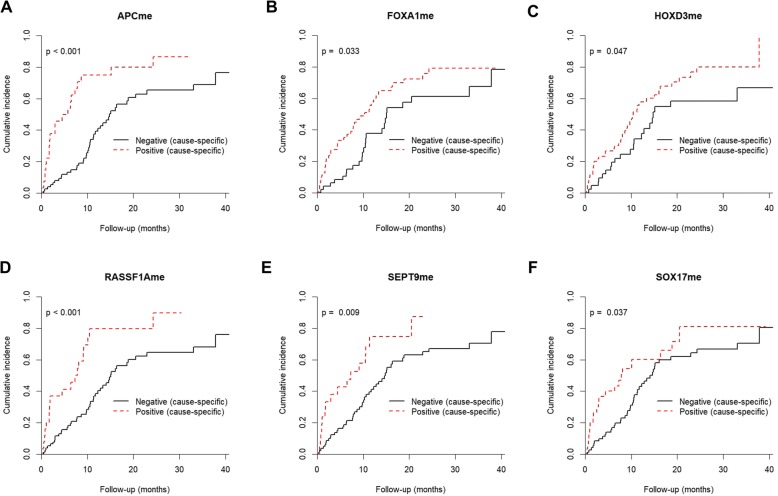
Cumulative incidence function plots according to **a** *APC*, **b** *FOXA1*, **c** *RASSF1A*, **d** *SEPT9* and **e** *SOX17* promoter methylation status in LC patients. Dashed red line and full black line represent positive and negative for promoter methylation status, respectively. *p* values obtained by Gray’s test for disease-specific mortality

Cumulative incidence of disease-specific mortality (DSM) was significantly increased in LC patients with lymph node involvement or distant metastasis (both *p* < 0.001), advanced clinical stage (*p* < 0.001) and SCLC subtype (*p* = 0.004) (Additional file 1: Table S4; Additional file 2: Figure S2), as expected. Interestingly, methylation of *APC* (*p* < 0.001), *FOXA1* (*p* = 0.033), *HOXD3* (*p* = 0.047), *RASSF1A* (*p* < 0.001), *SEPT9* (*p* = 0.009) and *SOX17* (*p* = 0.037) also significantly associated with increased cumulative incidence of DSM (Fig. 8 and Additional file 1: Table S5).

**Fig. 9:**
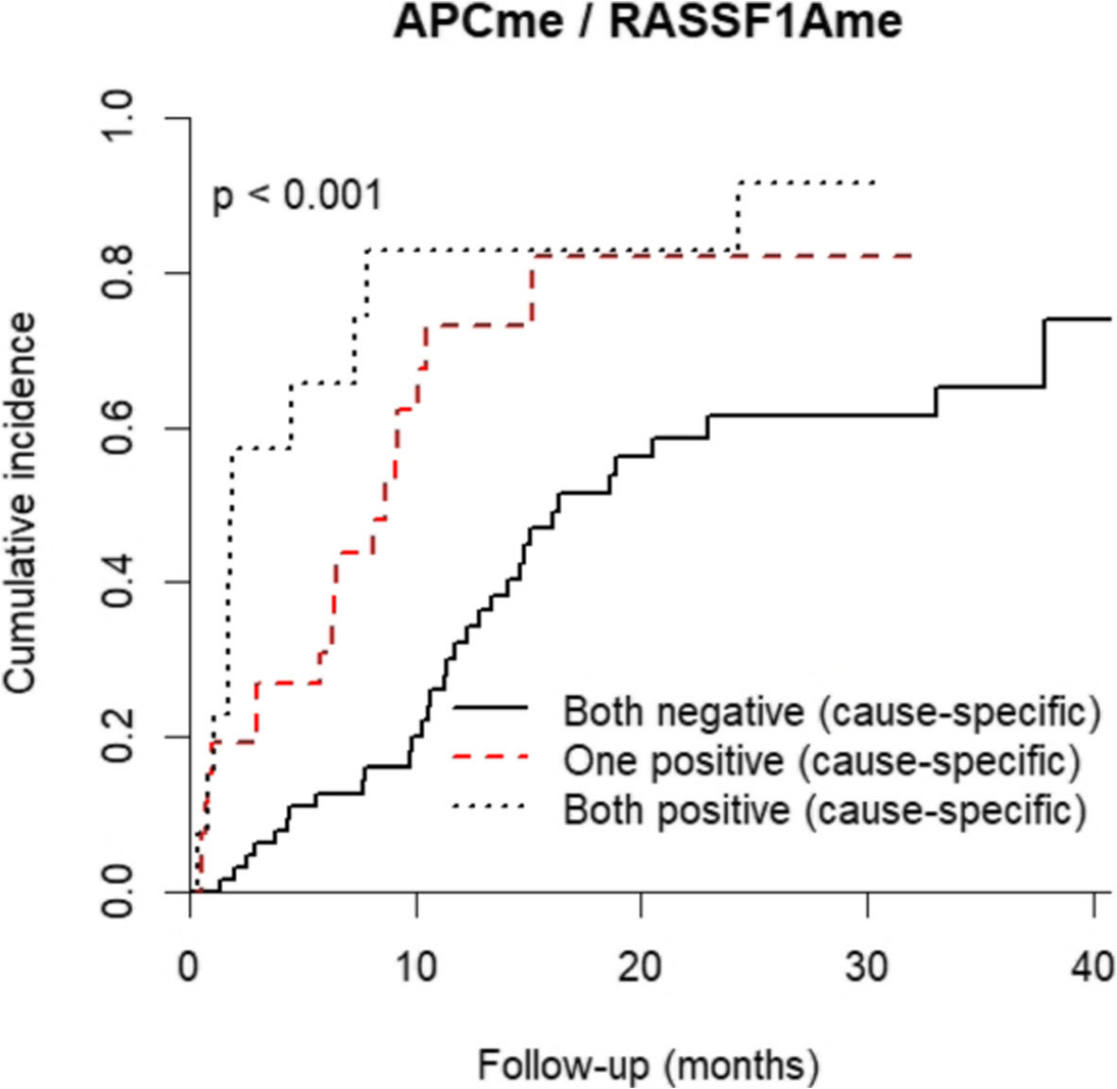
Cumulative incidence function plots according to panel (*APC*_*me*_ and *RASSF1A*_*me*_) promoter methylation status in LC patients. *p* values obtained by Gray’s test for disease-specific mortality

As both *APC* and *RASSF1A* methylation positivity were strongly associated with increased cumulative incidence of DSM, the value as a prognostic panel to better stratify outcome in LC patients was assessed for the combined genes. Indeed, this prognostic panel was also significantly associated with cumulative incidence of DSM (*p* < 0.001). LC patients classified as negative for methylation for the both genes presented an estimated 32% of cumulative incidence of DSM at 12 months, whereas the patients with only one methylated gene promoter presented a DSM cumulative incidence of 73%. Remarkably, the estimated DSM cumulative increased to 82% in patients classified as positive for both genes (Additional file 1: Table S4; Fig. 9).

On univariable Cox-regression analysis, circulating *APC*_*me*_ (*p* < 0.0001), *FOXA1*_*me*_ (*p* = 0.039), *RASSF1A*_*me*_ (*p* = 0.001), *SEPT9*_*me*_ (*p* = 0.018) and prognostic panel (*APC*_*me*_ and *RASSF1A*_*me*_) (both negative vs one positive and both negative vs both positive) (*p* < 0.0001) were DSM predictors in LC patients, along with nodal involvement (*p* = 0.001) and distant metastasis (*p* < 0.0001) at diagnosis, clinical stage (*p* = 0.001) and histological subtype (*p* = 0.015) (Table 6). Remarkably, on multivariable Cox-regression analysis comprising the significant variables in univariable analysis (clinical stage, histological subtype, *FOXA1*_*me*_, *SEPT9*_*me*_ and prognostic panel (*APC*_*me*_ and *RASSF1A*_*me*_)), both clinical stage (*p* = 0.002) and prognostic methylation panel (*APC*_*me*_ and *RASSF1A*_*me*_) (*p* < 0.0001) were found to be independent disease-specific mortality predictors (Table 6). Indeed, LC patients with detectable circulating *APC*_*me*_ and *RASSF1A*_*me*_ displayed a 3.918-fold risk (95%CI for HR 1.935–7.933, *p* < 0.0001) of dying from LC comparing to those lacking methylation (Table 6).

**Table 6:** Cox-regression models assessing the potential of clinical variables and detection of circulating *APC*_*me*_*, FOXA1*_*me*_*, HOXD3*_*me*_*, RASSF1A*_*me*_*, SEPT9*_*me*_*, SOX17*_*me*_ and prognostic panel (*APC*_*me*_*, RASSF1A*_*me*_) in the prediction of disease-specific mortalityin LC patients

Disease-Specific Mortality	Variable	HR	95% CI for HR	*P* value
Univariable	Regional node (N)			
	N0 *vs* N+	4.067	1.827-9.051	0.001
	Distant Metastasis (M)			
	M0 *vs* M+	3.320	1.897-5.809	<0.0001
	Clinical Stage			
	I&II *vs* III&IV	5.594	2.012-15.549	0.001
	Histological Subtype			
	NSCLC *vs* SCLC	2.098	1.153-3.819	0.015
	*APC*_*me*_			
	Negative *vs* positive	2.800	1.634-4.800	<0.0001
	*FOXA1*_*me*_			
	Negative *vs* positive	1.716	1.027-2.867	0.039
	*HOXD3*_*me*_			
	Negative *vs* positive	1.666	0.982-2.828	0.058
	*RASSF1A*_*me*_			
	Negative *vs* positive	2.669	1.529-4.660	0.001
	*SEPT9*_*me*_			
	Negative *vs* positive	2.011	1.130-3.579	0.018
	*SOX17*_*me*_			
	Negative *vs* positive	1.696	0.991-2.902	0.054
	*APC*_*me*_ */ RASSF1A*_*me*_			<0.0001
	Both negative *vs* One positive	2.582	1.428-4.671	0.002
	Both negative *vs* Both Positive	3.958	1.967-7.965	<0.0001
Multivariable	Clinical Stage			
	I&II *vs* III&IV	5.014	1.781-14.114	0.002
	*APC*_*me*_ */ RASSF1A*_*me*_			<0.0001
	Both negative *vs* One positive	1.992	1.100-3.608	0.023
	Both negative *vs* Both positive	3.918	1.935-7.933	<0.0001

PCa patient’s cohort displayed the longer follow-up time, with a median of 85 months (range, 3 to 155 months). From the 85 patients treated with curative intent (radical prostatectomy, external beam radiotherapy or brachytherapy), 24 developed biochemical relapse and 12 endured disease progression. Moreover, nine out of the remainder 36 patients (subjected to androgen deprivation therapy or active surveillance) presented disease progression. Nonetheless, no relevant significant associations were disclosed between circulating methylation positivity and PFS or DFS (data not shown). Although 38 patients deceased during follow-up, only 11 deaths were attributable to PCa. Cumulative incidence of DSM was significantly increased in PCa patients with higher serum PSA levels at diagnosis (*p* < 0.001), higher group grade (*p =* 0.007) and advanced clinical stage (*p* = 0.003). Interestingly, patients with detectable circulating *APC*_*me*_ (*p* < 0.001), *GSTP1*_*me*_ (*p* = 0.003), *RARβ2*_*me*_ (*p* = 0.001), *RASSF1A*_*me*_, *SEPT9*_*me*_ (both *p* < 0.001) or *SOX17*_*me*_ (*p* = 0.014) also disclosed increased cumulative incidence of DSM (Additional file 1: Table S5; Additional file 2: Figure S3). Nevertheless, Cox-regression analysis was not performed due to the reduced number of events in each group.

Regarding CRC patients, a median follow-up time of 13 months (range from 0 to 44 months) was achieved. From the 90 CRC patients submitted to surgery, 10 developed recurrence/progression. Given the difficulty to ascertain whether patients submitted to surgery were indeed free of the disease, DFS was not performed. CRC was the cause of death of nine of the 11 patients that deceased during follow-up. The presence of distant metastasis (*p* < 0.001) and detection of circulating *RARβ2*_*me*_ (*p* = 0.002), *SEPT9*_*me*_ (*p* < 0.001) and *SOX17*_*me*_ (*p* = 0.031) significantly associated with increased cumulative incidence of DSM (Additional file 1: Table S6; Additional file 2: Figure S4), although due to lack of events, no Cox-regression analysis was performed.

## Discussion

LC, PCa and CRC are the three most incident and prevalent cancers in males worldwide, and the most deadly in Europe and North America [1]. Despite efforts to develop more effective screening methods, due to either low sensitivity, high false-positive rate leading to overdiagnosis [7, 21, 22] or high invasiveness and cost [9], currently available strategies are suboptimal for population-based screening. Thus, development of new minimally invasive and effective pre-screening methods to triage patients for subsequent screening/detection tests are urgently needed. Aberrant DNA methylation of cancer-related genes occurs at very early stages of tumorigenesis, is cancer-specific, and amenable to be assessed in ccfDNA [13, 23], representing a valuable candidate tool for minimally invasive early cancer detection. Thus, we assessed the clinical utility of a liquid biopsy-based strategy for simultaneous detection of LC, PCa and CRC in males using multiplex qMSP in ccfDNA.

The eight candidate genes tested were selected based on a critical analysis of published data available in PubMed, including previously published data by our research team [14, 20, 24]. Firstly, the performance of candidate genes was individually analysed for each cancer type. In accordance with previously published studies [20, 25–33], significantly higher circulating *APC*_*me*_*, FOXA1*_*me*_*, RARβ2*_*me*_*, RASSF1A*_*me*_*, SEPT9*_*me*_ and *SOX17*_*me*_ levels were observed in LC patients. Although the best panel for LC detection—*FOXA1me, RARβ2*_me,_ *RASSF1A*_*me*_*, SOX17*_*me*_—disclosed similar specificity to “Epi proLung”, it showed modest sensitivity [16]. Concerning PCa, except for *APC*_*me*_ that was only reported in tissue and urine of cancer patients [34, 35], our results are in line with previously published studies showing *GSTP1*_*me*_, *RARβ2*_me_ and *RASSF1A*_*me*_ hypermethylation in ccfDNA of PCa patients [36–39]. Furthermore, to the best of our knowledge, we are the first research team reporting the biomarker potential of *FOXA1*_*me*_*, HOXD3*_*me*_*, SOX17*_*me*_ and *SEPT9*_*me*_ levels for PCa detection in blood-based liquid biopsies. Interestingly, the combination of *FOXA1*_*me*_*, RARβ2*_*me*_*, RASSF1A*_*me*_ and *GSTP1*_*m*_ identified PCa with 72% sensitivity and specificity. Although a panel comprising *RARβ2*_*me*_*, RASSF1A*_*me*_ and *GSTP1*_*me*_ disclosing 29% sensitivity and 100% specificity to detect PCa was previously shown [38], the addition of *FOXA1* to this panel in our study increased sensitivity although at expense of lower specificity. Nonetheless, the former panel was assessed using MSP [38], whereas we performed multiplex qMSP, which requires less amounts of DNA, is less time-consuming and more sensitive. We also confirmed *SEPT9* and *SOX17* hypermethylation in ccfDNA of CRC patients, although displaying modest sensitivity (12%), in contrast with previous reports [20, 40]. Moreover, the disparate results observed for *APC*_*me*_ *FOXA1*_*me*_, *RARβ2*_*me*_ and *RASSF1A*_*me*_ [20, 41–45] might be due to differences in sample collection procedures, methodology or population studied, since we have only evaluated these genes in males. Interestingly, *HOXD3* was significantly hypomethylated in CRC samples.

Although the initial goal was to identify a panel suitable for simultaneous detection of LC, PCa and CRC in males, due to the results observed in CRC, we decided to move forward with simultaneous detection of LC and PCa, only, which constitute the two most incident cancers in males. The best combination to this end—“PanCancer” panel (*FOXA1*_*me*_*, RARβ2*_*me*_ and *RASSF1A*_*me*_)—disclosed 70% specificity and 64% sensitivity, which is slightly lower than we have reported for the simultaneous detection of breast cancer (BrC), CRC and LC in women [20]. Nonetheless, compared to LD-CT LC screening [2], our test displayed considerable less false-positive results. Although asymptomatic donors included in our study do not represent a high-risk population, they might better reproduce the “real world” setting, nonetheless. Furthermore, the panel performance for detecting PCa, outperformed serum PSA specificity (which is around 20–45%) [46], although with limited sensitivity. Interestingly, our results are within the range reported for urinary long-non coding RNA Prostate Cancer Antigen 3 (PCA3), which is analysed in urine collected after prostatic massage [46, 47], a more invasive PCa screening approach. Thus, we are tempted to speculate whether our panel might add clinical value to serum PSA, increasing specificity and potentially reducing the number of unnecessary prostate biopsies, a hypothesis that deserves further studies.

Despite the modest sensitivity to detect early stage (I and II) LC, the high specificity of “PanCancer” panel to detect those patients suggests a potential usefulness for early detection of LC, reducing false-positive rates of some previously reported screening strategies. Moreover, our panel also discriminated intermediate and high-risk PCa (stage II-IV), according to the EAU risk groups [48], with 71% sensitivity and 65% specificity, thus increasing the ability to identify clinically significant PCa. Therefore, it has the potential to prevent the excess morbidity associated with unnecessary biopsies and reduce overdiagnosis and consequent overtreatment. Hence, the minimally invasive “PanCancer” panel might be advantageous for population-based screening, limiting drawbacks of currently available methods, with the advantage of potentially detecting two cancer types in the same test.

Following a positive result in “PanCancer” panel, identifying the primary location (i.e. LC or PCa) is of foremost importance. We, thus, propose a second panel (“CancerType”) comprising *SOX17*_*me*_ and *GSTP1*_*me*_, due to their remarkable specificity to discriminate between LC and PCa. Nonetheless, given the limited sensitivity of these two genes, this strategy must be complemented with other ancillary tests, including imaging. Notwithstanding, a positive “PanCancer” test should always trigger a search for LC or PCa and if any of these is identified, then, closer follow-up and repeat testing must be performed.

Correlations between clinicopathological features and circulating methylation levels of candidate genes were also assessed. Interestingly, higher *FOXA1*_*me*_ and *RARβ2*_*me*_ associated with advanced LC tumour stage. Moreover, methylation of those two genes, as well as of *APC*_*me*_ and *HOXD3*_*me*_, was also increased in node-positive disease. Furthermore, higher *APC*_*me*_*, FOXA1*_*me*_*, HOXD3*_*me*_ and *RASSF1A*_*me*_ associated with metastatic dissemination. To the best of our knowledge, only *RASSF1A*_*me*_ and *SOX17*_*me*_ have been previously associated with nodal and distant metastasis and advanced stage in ccfDNA of LC patients [20, 49]. Increased *HOXD3*_*me*_ levels were similarly observed in all five PCa patients with distant metastasis at diagnosis, along with higher *APC*_*me,*_ *GSTP1*_*me*_*, RARβ2*_*me*_*, RASSF1A*_*me*_ and *SEPT9*_*me*_ in four out of the same five patients. Likewise, higher *RARβ2*_*me*_*, SEPT9*_*me*_ and *SOX17*_*me*_ levels were disclosed in metastatic CRC patients, paralleling recent reports which indicate the same trend for *SEPT9*_*me*_ and *SOX17*_*me*_ [20, 50, 51]. Thus, despite the early onset of epigenetic alterations in tumorigenesis, higher circulating methylation levels are detectable in cancer progression probably owing to increased tumour burden and metastatic spread.

Remarkably, a panel constituted by *HOXD3*_*me*_ and *RASSF1A*_*me*_ was able to discriminate SCLC from NSCLC with 75% sensitivity and 88% specificity. Interestingly, *RASSF1A*_*me*_ potential for SCLC discrimination from NSCLC in plasma samples was previously observed in our group [52]. This distinction is critical as SCLC and NSCLC require quite different therapeutic strategies and display different prognosis. Although, an IVD-approved miRNA-based test (miRview) identifies the four main LC subtypes, in fine-needle aspirates and cytological samples, with 90% sensitivity and specificity [53], our panel can discriminated two major LC subtypes in liquid biopsies, which has a far less invasive collection method. Moreover, our panel displays similar performance to a three miRNAs panel assessed in plasma [54].

We further evaluated whether gene methylation might convey independent prognostic information. Indeed, detection of circulating *APC*_*me*_*, FOXA1*_*me*_*, HOXD3*_*me*_*, RASSF1A*_*me*_*, SEPT9*_*me*_ and *SOX17*_*me*_ associated with increased cumulative incidence of DSM in LC patients, alongside with all clinicopathological variables except tumour stage (T). Remarkably, the prognostic panel (*APC*_*me*_ and *RASSF1A*_*me*_) is an independent predictor of DSM in LC patients. These results are in line with the prognostic value reported for circulating *APC*_*me*_ and *RASSF1A*_*me*_ by other research teams, namely as biomarkers for evaluation of treatment efficacy and disease monitoring [26, 55, 56]. Moreover, detectable circulating *APC*_*me*_, *GSTP1*_*me*_, *RARβ2*_*me*_, *RASSF1A*_*me*_, *SEPT9*_*me*_ and *SOX17*_*me*_ methylation, in PCa patients, and *RARβ2*_*me*_, *SEPT9*_*me*_ and *SOX17*_*me*_, in CRC patients, associated with increased cumulative incidence of DSM, suggesting that methylation of selected genes in plasma might convey prognostic information already at the time of diagnosis. Our observations are limited owing to the short follow-up time and the favourable prognosis of most PCa and CRC patients, and, thus, additional validation in larger studies with longer follow-up is warranted.

Overall and notwithstanding the promising results of “PanCancer” panel for simultaneous detection of LC and PCa in males, it should be acknowledged that the lack of clinical information and long-term follow-up of asymptomatic controls of our exploratory study constitutes a limitation. Therefore, the screening utility of “PanCancer” panel in general population, as well as, in high-risk populations, such as high-risk smokers and family history of PCa, remains to be ascertained. Hence, our results should be validated in future large multicentre prospective studies. Nonetheless, one may envisage that high-risk populations could benefit from the implementation of this minimally invasive screening test given the lack of widely implemented options able to detect LC and PCa at early stages in these population subsets. Moreover, further studies should also be considered to find the best DNA methylation-based test to detect CRC, and ultimately, to be able to detect the four major cancers in both genders (LC, BrC, CRC and PCa).

## Conclusions

In conclusion, our findings corroborate the hypothesis that a single minimally invasive test can be devised to simultaneously detect multiple malignancies, which might improve patient compliance and, thereby, increase tumours’ detection rate at earlier stages, reducing cancer morbidity and mortality. Moreover, we also provide a novel tool to aid discrimination between the major LC subtypes and a prognostic panel that carries independent information for those patients.

## Material and methods

### Patients and sample collection

A case control retrospective cohort of LC, PCa and CRC male patients retrieved from consecutive series was included in this study. A total of 323 male patients diagnosed with LC (*n* = 102) or CRC (*n* = 100) between 2015 and 2019, and PCa (*n* = 121) between 2006 and 2010, at Portuguese Oncology Institute of Porto, Portugal, without previous oncological treatment were selected. For control purposes, blood samples were donated from 2016 to 2018 by 136 male asymptomatic blood donors (AC), without any known malignancy at the same institution.

After collection of peripheral blood into EDTA-containing tubes, plasma was separated by centrifuging at 2000 rpm for 10 min at 4 °C, and subsequently, stored at − 80 °C in the institutional tumour bank until further use. Relevant clinical and pathological data was retrieved from clinical charts and an anonymized database was constructed for analysis purposes.

### CcfDNA extraction, sodium-bisulfite modification and whole genome amplification

CcfDNA was extracted from 2 to 3 mL of plasma using QIAmp MinElute ccfDNA (Qiagen, Hilden, Germany), according to manufacturer’s instructions, and subsequently eluted in 20 μL of sterile distilled water and stored at − 20 °C until further use. Then, 20 μL of each extracted ccfDNA sample and 1 μg of CpGenome™ Universal Methylated DNA (Merck Milipore, Burlington, MA, USA) were sodium-bisulfite-modified using EZ DNA Methylation-GoldTM Kit (Zymo Research, Irvine, CA, USA) according to the manufacturer’s recommendations. Afterwards, the bisulfite-modified DNA was eluted in 10 μL of sterile distilled water and stored at − 80 °C until further use. EpiTect Whole Bisulfitome Kit (Qiagen, Hilden, Germany) was used to perform Whole Genome Amplification (WGA) of 10 μL sodium-bisulfite-modified DNA following manufacturer’s recommendations. Lastly, amplified DNA samples were diluted in 25 μL of sterile distilled water, in a final volume of 65 μL, and stored at − 20 °C until further use. Extracted, sodium-bisulfite converted and amplified DNA were quantified using Qubit 2 Fluorometer (Invitrogen, Carlsbad, CA, USA), according to manufacturer’s instructions.

### Multiplex qMSP

Promoter methylation levels of eight genes (*APC*_*me*_*, FOXA1*_*me*_*, GSTP1*_*me*_*, HOXD3*_*me*_*, RARβ2*_*me*_*, RASSF1A*_*me*_*, SEPT9*_*me*_ and *SOX17*_*me*_) were assessed by multiplex quantitative methylation-specific PCR (qMSP), using WGA-amplified DNA as template and Xpert Fast Probe (GRiSP, Porto, Portugal). Primers and TaqMan probes designed specifically for the modified gene sequence plus fluorochromes and quenchers selected for each probe are listed in Additional file 1: Table S7. The housekeeping gene *β-Actin* was used an internal reference gene to normalize the assay. Multiplex qMSP assay was carried out in triplicate using a 7500 Sequence Detector (Applied Biosystems, Perkin Elmer, CA, USA). Sterile distilled water was used as negative control in all plates. WGA-amplified CpGenome™ Universal Methylated DNA subjected to six serial dilutions (5x factor dilution) was used to generate a standard curve in each plate, allowing for relative quantification and PCR efficiency evaluation. All plates displayed efficiency values above 90%. Relative methylation levels were calculated as the ratio between the mean methylation levels of each gene and the respective value for *β*-Actin, multiplied by 1000, for easier tabulation.

### Statistical analysis

Non-parametric tests were performed to compare methylation levels of each gene promoter and to evaluate associations with clinicopathological features. Mann-Whitney U test was used for comparisons between two groups, while Kruskal-Wallis test was used for multiple groups, followed by Mann-Whitney *U* test with Bonferroni’s correction for pairwise comparisons. Scatter dot plots were constructed using a log10 scale in *y* axis. Spearman non-parametric test was performed to assess correlations between methylation levels and patients’ age. A result was considered statistically significant when *p value* < 0.05.

For each gene’s promoter, samples were categorized as methylated or non-methylated based on the cut-off values established using Youden’s *J* index (value combining highest sensitivity and specificity), through receiver operator characteristic (ROC) curve analysis [57], and areas under the curve (AUC) were calculated. When more than one value fulfilled this condition, the cut-off value allowing for higher sensitivity was chosen. Validity estimates (sensitivity, specificity, accuracy and positive (LR+) and negative (LR–) likelihood ratios) with 95% confidence intervals (CI) were determined to assess detection biomarker performance.

To improve detection performance of the selected genes, panels were constructed considering a positive result whenever at least one gene promoter was plotted as methylated in individual analysis, favouring gene combinations allowing maximum sensitivity. For “PanCancer” panel, validity estimates were calculated by joining LC and PCa (*n* = 223) vs AC samples (*n* = 136), whereas for “CancerType” panel, these were calculated by comparing one tumour type to the other. Validity estimates for the two panels were assessed by constructing multiple ROC curves via resampling analysis [58]. In short, samples were randomly divided into training (70%) and validation (30%) sets. Then, the cut-off value obtained combining the highest sensitivity and specificity in the training set, was used to calculate validity estimates in the validation set. This procedure was repeated 1000 times, and the mean value of sensitivity, specificity, accuracy, LR+ and LR– was computed.

Using a competing risk approach, cumulative incidence function (CIF) plots were constructed, and Gray’s test was used to test differences in disease-specific mortality (DSM) between groups considering clinicopathological variables and categorized gene promoter methylation status (positive: relative methylation levels > 0, and negative: relative methylation levels = 0). DSM was calculated as the time between the date of diagnosis and the date of cancer-related death. Kaplan-Meier curves were constructed, and log-rank test was used to compare progression-free survival (PFS) and disease-free survival (DFS) between groups, considering clinicopathological variables and categorized gene promoter methylation. PFS was calculated as the time between the date of diagnosis and the date of the first imaging exam showing disease progression, whereas DFS as the time between the date of curative intent treatment and the date of the first imaging exam showing disease progression or biochemical recurrence (for PCa). Cox proportional hazards regression was employed to calculate hazard ratios (HR) and 95% CI. Backwards conditional multivariable Cox-regression model comprising all significant variables on univariable analysis was computed to determine whether genes’ promoter methylation status was independently associated with DSM.

Two-tailed *p* values calculation, ROC curve analysis and PFS and DFS survival analysis and cox-regression analysis were performed using SPSS 25.0 for MacOS software (IBM-SPSS Inc., Chicago, IL, USA). Multiple ROC curves via resampling analysis and CIF analysis were performed using R v.3.4.4 (Vienna, Austria). Scatter dot plots were assembled using GraphPad Prism 7.0a for MacOS Software (GraphPad Software Inc., LA Jolla, CA, USA).

## Supplementary information

**Additional file 1: Table S1**. Associations between lung cancer patients’ clinicopathological features and *APC*, *FOXA1*, *GSTP1*, *HOXD3*, *RARβ2*, *RASSF1A*, *SEPT9* and *SOX17* promoters’ methylation levels. *p*-values obtained by Mann-Whitney U Test. **Table S2**. Associations between prostate cancer patients’ clinicopathological features and *APC, FOXA1, GSTP1, HOXD3, RARβ2, RASSF1A, SEPT9* and *SOX17* promoters’ methylation levels. *p*-values obtained by Mann-Whitney U Test for Primary Tumour (T) and Distant Metastasis (M), and by Kruskal-Wallis Test for Grade Group (GG), serum PSA levels and Clinical Stage. **Table S3**. Associations between colorectal cancer patients’ clinicopathological features and *APC, FOXA1, GSTP1, HOXD3, RARβ2, RASSF1A*, *SEPT9* and *SOX17* promoters’ methylation levels. *p*-values obtained by Mann-Whitney U Test for Primary Tumour (T), Regional Node (N), Distant Metastasis (M) and Clinical Stage, and by Kruskal-Wallis Test for Tumour Location. **Table S4**. Demographics of the clinicopathological features and *APC, FOXA1, GSTP1, HOXD3, RARβ2, RASSF1A, SEPT9* and *SOX17* promoters’ methylation levels in lung cancer patients, and their association with disease-specific mortality. *p*-values obtained by Gray’s test. **Table S5**. Demographics of the clinicopathological features and *APC, FOXA1, GSTP1, HOXD3, RARβ2, RASSF1A, SEPT9* and *SOX17* promoters’ methylation levels in prostate cancer patients, and their association with disease-specific mortality. *p*-values obtained by Gray’s test. **Table S6**. Demographics of the clinicopathological features and *APC, FOXA1, GSTP1, HOXD3, RARβ2, RASSF1A, SEPT9* and *SOX17* promoters’ methylation levels in colorectal cancer patients, and their association with disease-specific mortality. *p*-values obtained by Gray’s test. **Table S7**. Primers and probes sequences with respective fluorochrome and quencher.

**Additional file 2: Figure S1**. Distribution of methylation levels in PCa patients according with metastatic dissemination. (A) *APC*, (B) *GSTP1*, (C) *HOXD3*, (D) *RARβ2*, (E) *RASSF1A* and (F) *SEPT9* promoter’s methylation levels between non-metastatic (M0) (n=116) and metastatic (M+) (n=5) PCa patients. Mann-Whitney U Test, ***p<0.001, ****p<0.0001. Red horizontal lines represent median methylation levels. **Figure S2**. Cumulative incidence function plots according to clinicopathological variables (A) regional node, (B) distant metastasis, (C) clinical stage and (D) histological subtype in LC patients. *p*-values obtained by Gray’s test for disease-specific mortality. **Figure S3**. Cumulative incidence function plots according to clinicopathological variables (A) ISUP Grade Group, (B) serum PSA levels, (C) clinical stage, and (D) *APC*, (E) *GSTP1*, (F) *RARβ2*, (G) *RASSF1A*, (H) *SEPT9*, (I) *SOX17* promoter methylation levels in PCa patients. *p*-values obtained by Gray’s test for disease-specific mortality. **Figure S4**. Cumulative incidence function plots according to clinicopathological variable (A) distant metastasis, and (B) *RARβ2*, (C) *SEPT9* and (D) *SOX17* promoter methylation levels in CRC patients. *p*-values obtained by Gray’s test for disease-specific mortality.

## Data Availability

All data generated or analysed during this study are included in this published article and its supplementary information files.

## Abbreviations

AC: Asymptomatic controls
*APC*_*me*_: Methylated APC regulator of WNT signalling pathway
CcfDNA: Circulating cell-free DNA
CI: Confidence interval
CIF: Cumulative incidence function
CRC: Colorectal cancer
DFS: Disease-free survival
DRE: Digital rectal examination
DSM: Disease-specific mortality
FOBT: Faecal occult blood test
*FOXA1*_*me*_: Methylated Fork-head box A1
*GSTP1*_*me*_: Methylated Glutathione S-transferase pi 1
*HOXD3*_*me*_: Methylated Homeobox D3
HR: Hazard ratio
LC: Lung cancer
LD-CT: Low-dose computed tomography
NSCLC: Non-small cell lung carcinoma
PCa: Prostate cancer
*PCA3*: Prostate Cancer antigen 3
PFS: Progression-free survival
PSA: Prostate-specific antigen
qMSP: Quantitative methylation-specific PCR
*RARβ2*_*me*_: Methylated Retinoic acid receptor beta 2
*RASSF1A*_*me*_: Methylated Ras association domain family 1 isoform A
SCLC: Small cell lung carcinoma
*SEPT9*_*me*_: Methylated Septin 9
*SOX17*_*me*_: Methylated SRY-box transcription factor 17
WGA: Whole genome amplification

## References

[1] Bray F, Ferlay J, Soerjomataram I, Siegel RL, Torre LA, Jemal A: Global cancer statistics 2018: GLOBOCAN estimates of incidence and mortality worldwide for 36 cancers in 185 countries. CA Cancer J Clin 2018. 10.3322/caac.21492 30207593

[2] National Lung Screening Trial Research T, Aberle DR, Adams AM, Berg CD, Black WC, Clapp JD, Fagerstrom RM, Gareen IF, Gatsonis C, Marcus PM et al: Reduced lung-cancer mortality with low-dose computed tomographic screening. N Engl J Med 2011, 365(5):395-409. 10.1056/NEJMoa1102873 PMC4356534 21714641

[3] De Koning H. VDAC, Ten Haaf K., Oudkerk M.: Effects of volume CT lung cancer screening: mortality results of the NELSON randomised-controlled population based trial. In: World Conference on Lung Cancer 2018. Toronto, Canada: Journal of Thoracic Oncology; 2018: S185.

[4] Balata H, Evison M, Sharman A, Crosbie P, Booton R (2019). CT screening for lung cancer: are we ready to implement in Europe?. Lung Cancer.

[5] Wu GX, Raz DJ (2016). Lung Cancer Screening. Cancer Treat Res.

[6] Hoffman RM (2011). Clinical practice. Screening for prostate cancer. N Engl J Med.

[7] Etzioni R, Penson DF, Legler JM, di Tommaso D, Boer R, Gann PH, Feuer EJ (2002). Overdiagnosis due to prostate-specific antigen screening: lessons from U.S. prostate cancer incidence trends. J Natl Cancer Inst.

[8] Rawson JB, Bapat B (2012). Epigenetic biomarkers in colorectal cancer diagnostics. Expert Rev Mol Diagn.

[9] Simon K (2016). Colorectal cancer development and advances in screening. Clin Interv Aging.

[10] Esteller M (2008). Epigenetics in cancer. N Engl J Med.

[11] Jeronimo C, Henrique R (2014). Epigenetic biomarkers in urological tumors: a systematic review. Cancer Lett.

[12] Ellinger J, Muller SC, Dietrich D (2015). Epigenetic biomarkers in the blood of patients with urological malignancies. Expert Rev Mol Diagn.

[13] Costa-Pinheiro P, Montezuma D, Henrique R, Jeronimo C (2015). Diagnostic and prognostic epigenetic biomarkers in cancer. Epigenomics.

[14] Constancio V, Barros-Silva D, Jeronimo C, Henrique R. Known epigenetic biomarkers for prostate cancer detection and management: exploring the potential of blood-based liquid biopsies. Expert Rev Mol Diagn. 2019:1–9. 10.1080/14737159.2019.1604224 30961397

[15] Payne SR (2010). From discovery to the clinic: the novel DNA methylation biomarker (m)SEPT9 for the detection of colorectal cancer in blood. Epigenomics.

[16] Weiss G, Schlegel A, Kottwitz D, Konig T, Tetzner R (2017). Validation of the SHOX2/PTGER4 DNA methylation marker panel for plasma-based discrimination between patients with malignant and nonmalignant lung disease. J Thorac Oncol.

[17] Liu L, Toung JM, Jassowicz AF, Vijayaraghavan R, Kang H, Zhang R, Kruglyak KM, Huang HJ, Hinoue T, Shen H (2018). Targeted methylation sequencing of plasma cell-free DNA for cancer detection and classification. Ann Oncol.

[18] Li W, Li Q, Kang S, Same M, Zhou Y, Sun C, Liu CC, Matsuoka L, Sher L, Wong WH (2018). CancerDetector: ultrasensitive and non-invasive cancer detection at the resolution of individual reads using cell-free DNA methylation sequencing data. Nucleic Acids Res.

[19] Kang S, Li Q, Chen Q, Zhou Y, Park S, Lee G, Grimes B, Krysan K, Yu M, Wang W (2017). CancerLocator: non-invasive cancer diagnosis and tissue-of-origin prediction using methylation profiles of cell-free DNA. Genome Biol.

[20] Nunes Sandra, Moreira-Barbosa Catarina, Salta Sofia, Palma de Sousa Susana, Pousa Inês, Oliveira Júlio, Soares Marta, Rego Licínio, Dias Teresa, Rodrigues Jéssica, Antunes Luís, Henrique Rui, Jerónimo Carmen (2018). Cell-Free DNA Methylation of Selected Genes Allows for Early Detection of the Major Cancers in Women. Cancers.

[21] Nanavaty P, Alvarez MS, Alberts WM (2014). Lung cancer screening: advantages, controversies, and applications. Cancer Control.

[22] Plumb AA, Halligan S (2015). Colorectal cancer screening. Semin Roentgenol.

[23] Kulis M, Esteller M (2010). DNA methylation and cancer. Adv Genet.

[24] Freitas M, Ferreira F, Carvalho S, Silva F, Lopes P, Antunes L, Salta S, Diniz F, Santos LL, Videira JF (2018). A novel DNA methylation panel accurately detects colorectal cancer independently of molecular pathway. J Transl Med.

[25] Usadel H, Brabender J, Danenberg KD, Jeronimo C, Harden S, Engles J, Danenberg PV, Yang S, Sidransky D (2002). Quantitative adenomatous polyposis coli promoter methylation analysis in tumor tissue, serum, and plasma DNA of patients with lung cancer. Cancer Res.

[26] Ponomaryova AA, Rykova EY, Cherdyntseva NV, Skvortsova TE, Dobrodeev AY, Zav'yalov AA, Bryzgalov LO, Tuzikov SA, Vlassov VV, Laktionov PP (2013). Potentialities of aberrantly methylated circulating DNA for diagnostics and post-treatment follow-up of lung cancer patients. Lung Cancer.

[27] Fujiwara K, Fujimoto N, Tabata M, Nishii K, Matsuo K, Hotta K, Kozuki T, Aoe M, Kiura K, Ueoka H (2005). Identification of epigenetic aberrant promoter methylation in serum DNA is useful for early detection of lung cancer. Clin Cancer Res.

[28] Powrozek T, Krawczyk P, Kucharczyk T, Milanowski J (2014). Septin 9 promoter region methylation in free circulating DNA-potential role in noninvasive diagnosis of lung cancer: preliminary report. Med Oncol.

[29] Yang Z, Qi W, Sun L, Zhou H, Zhou B, Hu Y (2019). DNA methylation analysis of selected genes for the detection of early-stage lung cancer using circulating cell-free DNA. Adv Clin Exp Med.

[30] Balgkouranidou I, Chimonidou M, Milaki G, Tsaroucha E, Kakolyris S, Georgoulias V, Lianidou E (2016). SOX17 promoter methylation in plasma circulating tumor DNA of patients with non-small cell lung cancer. Clin Chem Lab Med.

[31] Hsu HS, Chen TP, Hung CH, Wen CK, Lin RK, Lee HC, Wang YC (2007). Characterization of a multiple epigenetic marker panel for lung cancer detection and risk assessment in plasma. Cancer.

[32] Begum S, Brait M, Dasgupta S, Ostrow KL, Zahurak M, Carvalho AL, Califano JA, Goodman SN, Westra WH, Hoque MO (2011). An epigenetic marker panel for detection of lung cancer using cell-free serum DNA. Clin Cancer Res.

[33] Hulbert A, Jusue-Torres I, Stark A, Chen C, Rodgers K, Lee B, Griffin C, Yang A, Huang P, Wrangle J (2017). Early detection of lung cancer using DNA promoter hypermethylation in plasma and sputum. Clin Cancer Res.

[34] Moreira-Barbosa C, Barros-Silva D, Costa-Pinheiro P, Torres-Ferreira J, Constancio V, Freitas R, Oliveira J, Antunes L, Henrique R, Jeronimo C (2018). Comparing diagnostic and prognostic performance of two-gene promoter methylation panels in tissue biopsies and urines of prostate cancer patients. Clin Epigenetics.

[35] Zhao F, Olkhov-Mitsel E, Kamdar S, Jeyapala R, Garcia J, Hurst R, Hanna MY, Mills R, Tuzova AV, O'Reilly E (2018). A urine-based DNA methylation assay, ProCUrE, to identify clinically significant prostate cancer. Clin Epigenetics.

[36] Wu T, Giovannucci E, Welge J, Mallick P, Tang WY, Ho SM (2011). Measurement of GSTP1 promoter methylation in body fluids may complement PSA screening: a meta-analysis. Br J Cancer.

[37] Dumache R, Puiu M, Motoc M, Vernic C, Dumitrascu V (2014). Prostate cancer molecular detection in plasma samples by glutathione S-transferase P1 (GSTP1) methylation analysis. Clin Lab.

[38] Sunami E, Shinozaki M, Higano CS, Wollman R, Dorff TB, Tucker SJ, Martinez SR, Mizuno R, Singer FR, Hoon DS (2009). Multimarker circulating DNA assay for assessing blood of prostate cancer patients. Clin Chem.

[39] Ellinger J, Haan K, Heukamp LC, Kahl P, Buttner R, Muller SC, von Ruecker A, Bastian PJ (2008). CpG island hypermethylation in cell-free serum DNA identifies patients with localized prostate cancer. Prostate.

[40] Song L, Jia J, Peng X, Xiao W, Li Y (2017). The performance of the SEPT9 gene methylation assay and a comparison with other CRC screening tests: a meta-analysis. Sci Rep.

[41] Cassinotti E, Melson J, Liggett T, Melnikov A, Yi Q, Replogle C, Mobarhan S, Boni L, Segato S, Levenson V (2012). DNA methylation patterns in blood of patients with colorectal cancer and adenomatous colorectal polyps. Int J Cancer.

[42] Wang YC, Yu ZH, Liu C, Xu LZ, Yu W, Lu J, Zhu RM, Li GL, Xia XY, Wei XW (2008). Detection of RASSF1A promoter hypermethylation in serum from gastric and colorectal adenocarcinoma patients. World J Gastroenterol.

[43] Roperch JP, Incitti R, Forbin S, Bard F, Mansour H, Mesli F, Baumgaertner I, Brunetti F, Sobhani I (2013). Aberrant methylation of NPY, PENK, and WIF1 as a promising marker for blood-based diagnosis of colorectal cancer. BMC Cancer.

[44] Pack SC, Kim HR, Lim SW, Kim HY, Ko JY, Lee KS, Hwang D, Park SI, Kang H, Park SW (2013). Usefulness of plasma epigenetic changes of five major genes involved in the pathogenesis of colorectal cancer. Int J Color Dis.

[45] Lee BB, Lee EJ, Jung EH, Chun HK, Chang DK, Song SY, Park J, Kim DH (2009). Aberrant methylation of APC, MGMT, RASSF2A, and Wif-1 genes in plasma as a biomarker for early detection of colorectal cancer. Clin Cancer Res.

[46] Kearns JT, Lin DW (2018). Improving the specificity of PSA screening with serum and urine markers. Curr Urol Rep.

[47] Cui Y, Cao W, Li Q, Shen H, Liu C, Deng J, Xu J, Shao Q (2016). Evaluation of prostate cancer antigen 3 for detecting prostate cancer: a systematic review and meta-analysis. Sci Rep.

[48] Mottet N, Bellmunt J, Bolla M, Briers E, Cumberbatch MG, De Santis M, Fossati N, Gross T, Henry AM, Joniau S (2017). EAU-ESTRO-SIOG Guidelines on Prostate Cancer. Part 1: Screening, Diagnosis, and Local Treatment with Curative Intent. Eur Urol.

[49] Wang Y, Yu Z, Wang T, Zhang J, Hong L, Chen L (2007). Identification of epigenetic aberrant promoter methylation of RASSF1A in serum DNA and its clinicopathological significance in lung cancer. Lung Cancer.

[50] Fu B, Yan P, Zhang S, Lu Y, Pan L, Tang W, Chen S, Chen S, Zhang A, Liu W (2018). Cell-Free Circulating Methylated SEPT9 for Noninvasive Diagnosis and Monitoring of Colorectal Cancer. Dis Markers.

[51] Bergheim J, Semaan A, Gevensleben H, Groening S, Knoblich A, Dietrich J, Weber J, Kalff JC, Bootz F, Kristiansen G (2018). Potential of quantitative SEPT9 and SHOX2 methylation in plasmatic circulating cell-free DNA as auxiliary staging parameter in colorectal cancer: a prospective observational cohort study. Br J Cancer.

[52] Nunes SP, Diniz F, Moreira-Barbosa C, Constancio V, Silva AV, Oliveira J, Soares M, Paulino S, Cunha AL, Rodrigues J (2019). Subtyping Lung Cancer Using DNA Methylation in Liquid Biopsies. J Clin Med.

[53] Gilad S, Lithwick-Yanai G, Barshack I, Benjamin S, Krivitsky I, Edmonston TB, Bibbo M, Thurm C, Horowitz L, Huang Y (2012). Classification of the four main types of lung cancer using a microRNA-based diagnostic assay. J Mol Diagn.

[54] Lu S, Kong H, Hou Y, Ge D, Huang W, Ou J, Yang D, Zhang L, Wu G, Song Y (2018). Two plasma microRNA panels for diagnosis and subtype discrimination of lung cancer. Lung Cancer.

[55] Zhai X, Li SJ (2014). Methylation of RASSF1A and CDH13 genes in individualized chemotherapy for patients with non-small cell lung cancer. Asian Pac J Cancer Prev.

[56] Wang H, Zhang B, Chen D, Xia W, Zhang J, Wang F, Xu J, Zhang Y, Zhang M, Zhang L (2015). Real-time monitoring efficiency and toxicity of chemotherapy in patients with advanced lung cancer. Clin Epigenetics.

[57] Schisterman EF, Perkins NJ, Liu A, Bondell H (2005). Optimal cut-point and its corresponding Youden Index to discriminate individuals using pooled blood samples. Epidemiology.

[58] Baker SG, Kramer BS (2006). Identifying genes that contribute most to good classification in microarrays. BMC Bioinformatics.

