# Supplementary material for: Early detection of the major male cancer types in blood-based liquid biopsies using a DNA methylation panel
Source: Clin Epigenetics. 2019 Dec 2;11:175. doi: 10.1186/s13148-019-0779-x (PMC6889617; doi:10.1186/s13148-019-0779-x)
Supplement: Supplementary file 1 — Additional file 1: Table S1. Associations between lung cancer patients’ clinicopathological features and APC, FOXA1, GSTP1, HOXD3, RARβ2, RASSF1A, SEPT9 and SOX17 promoters’ methylation levels. p-values obtained by Mann-Whitney U Test. Table S2. Associations between prostate cancer patients’ clinicopathological features and APC, FOXA1, GSTP1, HOXD3, RARβ2, RASSF1A, SEPT9 and SOX17 promoters’ methylation levels. p-values obtained by Mann-Whitney U Test for Primary Tumour (T) and Distant Metastasis (M), and by Kruskal-Wallis Test for Grade Group (GG), serum PSA levels and Clinical Stage. Table S3. Associations between colorectal cancer patients’ clinicopathological features and APC, FOXA1, GSTP1, HOXD3, RARβ2, RASSF1A, SEPT9 and SOX17 promoters’ methylation levels. p-values obtained by Mann-Whitney U Test for Primary Tumour (T), Regional Node (N), Distant Metastasis (M) and Clinical Stage, and by Kruskal-Wallis Test for Tumour Location. Table S4. Demographics of the clinicopathological features and APC, FOXA1, GSTP1, HOXD3, RARβ2, RASSF1A, SEPT9 and SOX17 promoters’ methylation levels in lung cancer patients, and their association with disease-specific mortality. p-values obtained by Gray’s test. Table S5. Demographics of the clinicopathological features and APC, FOXA1, GSTP1, HOXD3, RARβ2, RASSF1A, SEPT9 and SOX17 promoters’ methylation levels in prostate cancer patients, and their association with disease-specific mortality. p-values obtained by Gray’s test. Table S6. Demographics of the clinicopathological features and APC, FOXA1, GSTP1, HOXD3, RARβ2, RASSF1A, SEPT9 and SOX17 promoters’ methylation levels in colorectal cancer patients, and their association with disease-specific mortality. p-values obtained by Gray’s test. Table S7. Primers and probes sequences with respective fluorochrome and quencher. [file 13148_2019_779_MOESM1_ESM.docx]

| **Genes** | ***APC_me_*** | ***FOXA1_me_*** | ***GSTP1_me_*** | ***HOXD3_me_*** | ***RARβ2_me_*** | ***RASSF1A_me_*** | ***SEPT9_me_*** | ***SOX17_me_*** |
| --- | --- | --- | --- | --- | --- | --- | --- | --- |
| **Clinicopathological features** | *p-*value | | | | | | | |
| **Histological type** | 0.162 | 0.516 | 0.379 | **0.001** | 0.338 | **<0.0001** | 0.532 | **0.013** |
| **Primary Tumour (T)** | 0.490 | **0.014** | 0.503 | 0.622 | **0.044** | 0.137 | 0.066 | 0.091 |
| **Regional Node (N)** | **0.044** | **0.021** | 0.302 | **0.022** | **0.015** | 0.085 | 0.381 | 0.471 |
| **Distant Metastasis (M)** | **0.028** | **0.001** | 0.655 | **<0.0001** | 0.323 | **0.001** | 0.179 | 0.119 |
| **Clinical Stage** | 0.080 | 0.228 | 0.434 | 0.784 | 0.141 | **0.043** | 0.277 | 0.469 |

**Supplementary Table 1.** Associations between lung cancer patients’ clinicopathological features and APC, FOXA1, GSTP1, HOXD3, RARβ2, RASSF1A, SEPT9 and SOX17 promoters’ methylation levels. p-values obtained by Mann-Whitney U Test.

| **Genes** | ***APC_me_*** | ***FOXA1_me_*** | ***GSTP1_me_*** | ***HOXD3_me_*** | ***RARβ2_me_*** | ***RASSF1A_me_*** | ***SEPT9_me_*** | ***SOX17_me_*** |
| --- | --- | --- | --- | --- | --- | --- | --- | --- |
| **Clinicopathological features** | *p-*value | | | | | | | |
| **Primary Tumour (T)** | 0.170 | 0.861 | 0.584 | 0.994 | 0.357 | 0.989 | 0.577 | 0.865 |
| **Distant Metastasis (M)** | **<0.0001** | 0.979 | **<0.0001** | **0.003** | **<0.0001** | **<0.0001** | **<0.0001** | 0.111 |
| **Grade Group (GG)** | 0.355 | 0.091 | 0.056 | 0.565 | 0.269 | 0.095 | 0.060 | 0.852 |
| **PSA** | 0.052 | 0.413 | 0.237 | 0.084 | 0.396 | 0.213 | **0.038** | 0.624 |
| **Clinical Stage** | **0.001** | 0.841 | **<0.0001** | 0.073 | **0.001** | **<0.0001** | **<0.0001** | 0.052 |

**Supplementary Table 2.** Associations between prostate cancer patients’ clinicopathological features and APC, FOXA1, GSTP1, HOXD3, RARβ2, RASSF1A, SEPT9 and SOX17 promoters’ methylation levels. p-values obtained by Mann-Whitney U Test for Primary Tumour (T) and Distant Metastasis (M), and by Kruskal-Wallis Test for Grade Group (GG), serum PSA levels and Clinical Stage. Regional Node (N) was not evaluated due to the low number of N+ samples.

| **Genes** | | ***APC_me_*** | ***FOXA1_me_*** | ***GSTP1_me_*** | ***HOXD3_me_*** | ***RARβ2_me_*** | ***RASSF1A_me_*** | ***SEPT9_me_*** | ***SOX17_me_*** |
| --- | --- | --- | --- | --- | --- | --- | --- | --- | --- |
| **Clinicopathological features** | | *p-*value | | | | | | | |
| **Tumour Location** | 0.898 | | 0.884 | 0.487 | 0.932 | 0.068 | 0.267 | 0.416 | 0.249 |
| **Primary Tumour (T)** | | 0.941 | 0.553 | 0.548 | 0.524 | 0.078 | 0.449 | 0.101 | 0.169 |
| **Regional Node (N)** | | 0.358 | 0.534 | 0.402 | 0.199 | 0.083 | 0.472 | 0.127 | 0.460 |
| **Distant Metastasis (M)** | | 0.306 | 0.521 | 0.639 | 0.723 | **<0.0001** | 0.059 | **<0.0001** | **<0.0001** |
| **Clinical Stage** | | 0.277 | 0.638 | 0.424 | 0.133 | **0.012** | 0.549 | **0.019** | 0.160 |

**Supplementary Table 3.** Associations between colorectal cancer patients’ clinicopathological features and *APC, FOXA1, GSTP1, HOXD3, RARβ2, RASSF1A*, *SEPT9* and *SOX17* promoters’ methylation levels. *p*-values obtained by Mann-Whitney U Test for Primary Tumour (T), Regional Node (N), Distant Metastasis (M) and Clinical Stage, and by Kruskal-Wallis Test for Tumour Location. Histological type was not evaluated due to the low number of squamous cell carcinoma samples.

|  | **Patients**  **n (%)** | **Cumulative Incidence Function (DSM)**  ***p*-value** |
| --- | --- | --- |
| **Tumour stage (T)^a^**  (T1, T2-4) | 12 (11.8%), 82 (80.4%) | 0.093 |
| **Regional node (N)^b^**  (N0, N+) | 25 (24.5%), 72 (70.6%) | **<0.001** |
| **Distant Metastasis (M)**  (M0, M+) | 47 (46.1%), 55 (53.9%) | **<0.001** |
| **Clinical Stage**  (I&II, III&IV) | 17 (16.7%), 85 (83.3%) | **<0.001** |
| **Histological Subtype**  (NSCLC, SCLC) | 86 (84.3%), 16 (15.7%) | **0.004** |
| ***APC_me_***  (Negative, positive) | 75 (73.5%), 27 (26.5%) | **<0.001** |
| ***FOXA1_me_***  (Negative, positive) | 47 (46.1%), 55 (53.9%) | **0.033** |
| ***GSTP1_me_***  (Negative, positive) | 99 (97.1%), 3 (2.9%) | 0.068 |
| ***HOXD3_me_***  (Negative, positive) | 42 (41.2%), 60 (58.8%) | **0.047** |
| ***RAR𝛽2_me_***  (Negative, positive) | 78 (76.5%), 24 (23.5%) | 0.204 |
| ***RASSF1A_me_***  (Negative, positive) | 77 (75.5%), 25 (24.5%) | **<0.001** |
| ***SEPT9_me_***  (Negative, positive) | 81 (79.4%), 21 (20.6%) | **0.009** |
| ***SOX17_me_***  (Negative, positive) | 72 (70.6%), 30 (29.4%) | **0.037** |
| ***APC_me_ / RASSF1A_me_***  (Both negative, One positive,  Both Positive) | 63 (61.8%), 26 (25.5%), 13 (12.7%) | **<0.001** |

^a^No information available in 8 cases (7.8%); ^b^No information available in 5 cases (4.9%).

**Supplementary Table 4.** Demographics of the clinicopathological features and *APC, FOXA1, GSTP1, HOXD3, RARβ2, RASSF1A, SEPT9* and *SOX17* promoters’ methylation levels in lung cancer patients, and their association with disease-specific mortality. *p*-values obtained by Gray’s test.

|  | **Patients**  **n (%)** | **Cumulative Incidence Function (DSM)**  ***p*-value** |
| --- | --- | --- |
| **Tumour stage (T)^a^**  (T1-2, T3) | 104 (86%), 16 (13.2%) | 0.837 |
| **Grade Group**  (1, 2, 3-5) | 59 (48.8%), 38 (31.4%), 24 (19.8%) | **0.007** |
| **PSA levels**  (<10, 10-20, <20) | 71 (58.7%), 27 (22.3%), 23 (19%) | **<0.001** |
| **Clinical Stage**  (I, II, III&IV) | 31 (25.6%), 55 (45.5%), 35 (28.9%) | **0.003** |
| ***APC_me_***  (Negative, positive) | 97 (80.2%), 24 (19.8%) | **<0.001** |
| ***FOXA1_me_***  (Negative, positive) | 31 (25.6%), 90 (74.4%) | 0.813 |
| ***GSTP1_me_***  (Negative, positive) | 103 (85.1%), 18 (14.9%) | **0.003** |
| ***HOXD3_me_***  (Negative, positive) | 24 (19.8%), 97 (80.2%) | 0.094 |
| ***RAR𝛽2_me_***  (Negative, positive) | 94 (77.7%), 27 (22.3%) | **0.001** |
| ***RASSF1A_me_***  (Negative, positive) | 105 (86.8%), 16 (13.2%) | **<0.001** |
| ***SEPT9_me_***  (Negative, positive) | 107 (88.4%), 14 (11.6%) | **<0.001** |
| ***SOX17_me_***  (Negative, positive) | 86 (71.1%), 35 (28.9%) | **0.014** |

^a^No information available in 1 case (0.8%); Regional Node (N) and Distant Metastasis (M) was not evaluated due to the low number of N+ and M+ samples.

**Supplementary Table 5.** Demographics of the clinicopathological features and *APC, FOXA1, GSTP1, HOXD3, RARβ2, RASSF1A, SEPT9* and *SOX17* promoters’ methylation levels in prostate cancer patients, and their association with disease-specific mortality. *p*-values obtained by Gray’s test.

|  | **Patients**  **n (%)** | **Cumulative Incidence Function (DSM)**  ***p*-value** |
| --- | --- | --- |
| **Tumour location**  (Proximal colon, Distal colon, Rectum) | 23 (23%), 36 (36%), 41 (41%) | 0.771 |
| **Tumour stage (T)^a^**  (T1/T2, T3/T4) | 26 (26%), 72 (72%) | 0.169 |
| **Regional node (N)^b^**  (N0, N+) | 40 (40%), 57 (57%) | 0.784 |
| **Distant Metastasis (M)**  (M0, M+) | 82 (82%), 18 (18%) | **<0.001** |
| **Clinical Stage**  (I&II, III&IV) | 39 (39%), 61 (61%) | 0.148 |
| ***APC_me_***  (Negative, positive) | 88 (88%), 12 (12%) | 0.266 |
| ***FOXA1_me_***  (Negative, positive) | 53 (53%), 47 (47%) | 0.124 |
| ***GSTP1_me_***  (Negative, positive) | 99 (99%), 1 (1%) | 0.809 |
| ***HOXD3_me_***  (Negative, positive) | 60 (60%), 40 (40%) | 0.251 |
| ***RAR𝛽2_me_***  (Negative, positive) | 91 (91%), 9 (9%) | **0.002** |
| ***RASSF1A_me_***  (Negative, positive) | 93 (93%), 7 (7%) | 0.402 |
| ***SEPT9_me_***  (Negative, positive) | 92 (92%), 8 (8%) | **<0.001** |
| ***SOX17_me_***  (Negative, positive) | 84 (84%), 16 (16%) | **0.031** |

^a^No information available in 2 cases (2%); ^b^No information available in 3 cases (3%).

**Supplementary Table 6.** Demographics of the clinicopathological features and *APC, FOXA1, GSTP1, HOXD3, RARβ2, RASSF1A, SEPT9* and *SOX17* promoters’ methylation levels in colorectal cancer patients, and their association with disease-specific mortality. *p*-values obtained by Gray’s test. Histological type was not evaluated due to the low number of squamous cell carcinoma samples.

| **Gene** | | **Sequences** |
| --- | --- | --- |
| ***𝛽-Actin*** | **Primers** | F – 5’ TGG TGA TGG AGG AGG TTT AGT AAG T 3’ |
|  |  | R – 5’ ACC AAT AAA ACC TAC TCC TCC CTT AA 3´ |
|  | **Probe** | 5’ Cy5 – ACC ACC ACC CAA CAC ACA ATA ACA AAC ACA – QSY 3’ |
| ***APC_me_*** | **Primers** | F – 5’ TGT GTT TTA TTG CGG AGT GC 3’ |
|  |  | R – 5’ CAC ATA TCG ATC ACG TAC GC 5’ |
|  | **Probe** | 5’ VIC – CAA TCG ACG AAC TCC CGA C – MGB 3’ |
| ***FOXA1_me_*** | **Primers** | F – 5’ CGA CGT TAA GAC GTT TAA GC 3’ |
|  |  | R – 5’ CGC TCA ACG TAA ACA TCT TAC 3’ |
|  | **Probe** | 5’ FAM – ATA TAC GAA TAA AAC GAC TTA ACG – MGB 3’ |
| ***GSTP1_me_*** | **Primers** | F – 5’ GTC GGC GTC GTA TTT AGT ATT G 3’ |
|  |  | R – 5’ AAA CTA CGA CGA CGA AAC TCC AA 3’ |
|  | **Probe** | 5’ FAM – AAA CCT CGC ACC TCC GAA CCT TAT AAA A – BHQ1 3’ |
| ***HOXD3_me_*** | **Primers** | F – 5’ TAA AGG TTT ATG GTT GCG C 3’ |
|  |  | R – 5’ TTA CGA ACA CTA AAC TAC ACC CG 3’ |
|  | **Probe** | 5’ Cy5 – ACA AAA CGT TCC CGA CGC TTC TAA AA – BHQ1 3’ |
| ***RAR𝛽2_me_*** | **Primers** | F – 5’ TCG AGA ACG CGA GCG ATT 3’ |
|  |  | R – 5’ GAC CAA TCC AAC CGA AAC 3’ |
|  | **Probe** | 5’ HEX – CTT ACA AAA AAC CTT CCG AAT ACG TTC CGA – Iowa Black RQ-Sp 3’ |
| ***RASSF1A_me_*** | **Primers** | F – 5’ AGC GAA GTA CGG GTT TAA TC 3’ |
|  |  | R – 5’ ACA CGC TCC AAC CGA ATA 3’ |
|  | **Probe** | 5’ NED – CGG GAG TTG GTA TTC GTT GGG CG – QSY 3’ |
| ***SEPT9_me_*** | **Primers** | F – 5’ TTA GTT AGC GCG TAG GGT TC 3’ |
|  |  | R – 5’ ACC TTC GAA ATC CGA AAT AA 3’ |
|  | **Probe** | 5’ NED – GCG TTA ACC GCG AAA TCC GAC ATA ATA ACT – QSY 3’ |

| ***SOX17_me_*** | **Primers** | F – 5’ GAT CGG TTC GTT TTC GTC G 3’ |
| --- | --- | --- |
|  |  | R – 5’ GCC CGT ATT CTA ACC TAT CG 3’ |
|  | **Probe** | 5’ Cy5 – ACC GAC CTA ATA ACA CTA CGA ACG C – Iowa Black RQ-Sp 3’ |

**Supplementary Table 7.** Primers and probes sequences with respective fluorochrome and quencher.
