# Supplementary material for: Early detection of the major male cancer types in blood-based liquid biopsies using a DNA methylation panel
Source: Clin Epigenetics. 2019 Dec 2;11:175. doi: 10.1186/s13148-019-0779-x (PMC6889617; doi:10.1186/s13148-019-0779-x)
Supplement: Supplementary file 2 — Additional file 2: Figure S1. Distribution of methylation levels in PCa patients according with metastatic dissemination. (A) APC, (B) GSTP1, (C) HOXD3, (D) RARβ2, (E) RASSF1A and (F) SEPT9 promoter’s methylation levels between non-metastatic (M0) (n=116) and metastatic (M+) (n=5) PCa patients. Mann-Whitney U Test, ***p<0.001, ****p<0.0001. Red horizontal lines represent median methylation levels. Figure S2. Cumulative incidence function plots according to clinicopathological variables (A) regional node, (B) distant metastasis, (C) clinical stage and (D) histological subtype in LC patients. p-values obtained by Gray’s test for disease-specific mortality. Figure S3. Cumulative incidence function plots according to clinicopathological variables (A) ISUP Grade Group, (B) serum PSA levels, (C) clinical stage, and (D) APC, (E) GSTP1, (F) RARβ2, (G) RASSF1A, (H) SEPT9, (I) SOX17 promoter methylation levels in PCa patients. p-values obtained by Gray’s test for disease-specific mortality. Figure S4. Cumulative incidence function plots according to clinicopathological variable (A) distant metastasis, and (B) RARβ2, (C) SEPT9 and (D) SOX17 promoter methylation levels in CRC patients. p-values obtained by Gray’s test for disease-specific mortality. [file 13148_2019_779_MOESM2_ESM.docx]

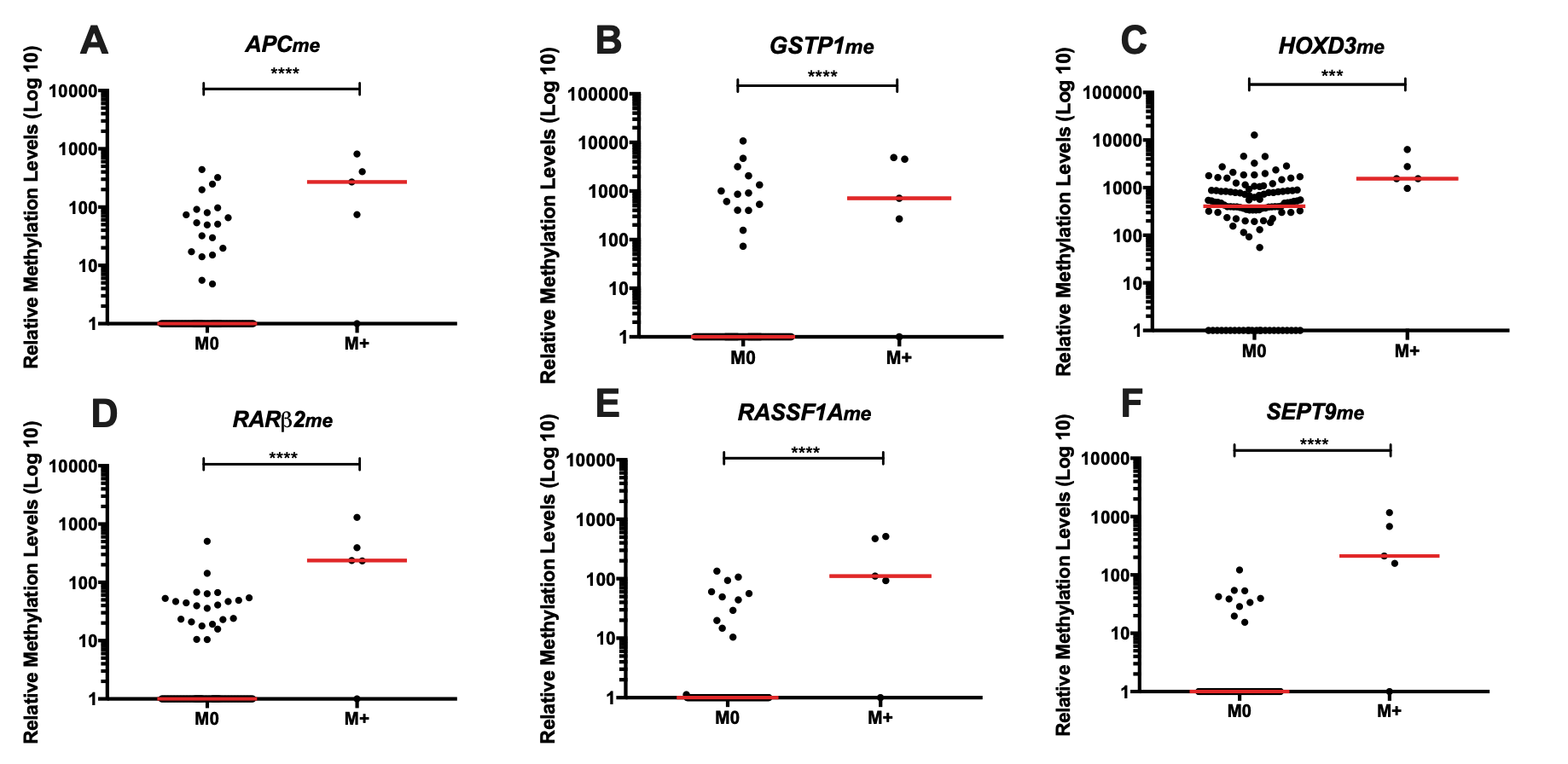


**Supplementary Figure 1.** Distribution of methylation levels in PCa patients according with metastatic dissemination. (A) *APC*, (B) *GSTP1*, (C) *HOXD3*, (D) *RARβ2*, (E) *RASSF1A* and (F) *SEPT9* promoter’s methylation levels between non-metastatic (M0) (n=116) and metastatic (M+) (n=5) PCa patients. Mann-Whitney U Test, ****p*<0.001, *****p*<0.0001. Red horizontal lines represent median methylation levels.

**Supplementary Figure 2**. Cumulative incidence function plots according to clinicopathological variables (A) regional node, (B) distant metastasis, (C) clinical stage and (D) histological subtype in LC patients. *p*-values obtained by Gray’s test for disease-specific mortality.

**Supplementary Figure 3.** Cumulative incidence function plots according to clinicopathological variables (A) ISUP Grade Group, (B) serum PSA levels, (C) clinical stage, and (D) *APC*, (E) *GSTP1*, (F) *RARβ2*, (G) *RASSF1A*, (H) *SEPT9*, (I) *SOX17* promoter methylation levels in PCa patients. *p*-values obtained by Gray’s test for disease-specific mortality.

**Supplementary Figure 4.** Cumulative incidence function plots according to clinicopathological variable (A) distant metastasis, and (B) *RARβ2*, (C) *SEPT9* and (D) *SOX17* promoter methylation levels in CRC patients. *p*-values obtained by Gray’s test for disease-specific mortality.
